# Estimation in meta‐analyses of mean difference and standardized mean difference

**DOI:** 10.1002/sim.8422

**Published:** 2019-11-11

**Authors:** Ilyas Bakbergenuly, David C. Hoaglin, Elena Kulinskaya

**Affiliations:** ^1^ School of Computing Sciences University of East Anglia Norwich UK; ^2^ Population and Quantitative Health Sciences University of Massachusetts Medical School Worcester Massachusetts

**Keywords:** between‐study variance, mean difference, meta‐analysis, random‐effects model, standardized mean difference

## Abstract

Methods for random‐effects meta‐analysis require an estimate of the between‐study variance, *τ* ^2^. The performance of estimators of *τ* ^2^ (measured by bias and coverage) affects their usefulness in assessing heterogeneity of study‐level effects and also the performance of related estimators of the overall effect. However, as we show, the performance of the methods varies widely among effect measures. For the effect measures mean difference (MD) and standardized MD (SMD), we use improved effect‐measure‐specific approximations to the expected value of *Q* for both MD and SMD to introduce two new methods of point estimation of *τ* ^2^ for MD (Welch‐type and corrected DerSimonian‐Laird) and one WT interval method. We also introduce one point estimator and one interval estimator for *τ* ^2^ in SMD. Extensive simulations compare our methods with four point estimators of *τ* ^2^ (the popular methods of DerSimonian‐Laird, restricted maximum likelihood, and Mandel and Paule, and the less‐familiar method of Jackson) and four interval estimators for *τ* ^2^ (profile likelihood, Q‐profile, Biggerstaff and Jackson, and Jackson). We also study related point and interval estimators of the overall effect, including an estimator whose weights use only study‐level sample sizes. We provide measure‐specific recommendations from our comprehensive simulation study and discuss an example.

## 1 INTRODUCTION

Meta‐analysis is a statistical methodology for combining estimated effects from several studies in order to assess their heterogeneity and obtain an overall estimate. In this paper, we focus on meta‐analysis of continuous outcomes. The data and, often, existing tradition determine the choice of outcome measure. In a comparative study with continuous subject‐level data for a treatment arm (T) and a control arm (C), the customary outcome measures are the mean difference (MD) and the standardized MD (SMD). Part 2, Chapter 9, of the *Cochrane Handbook* 1 pointed out that the choice between MD and SMD depends on whether “outcome measurements in all studies are made on the same scale.” However, fields of application have established preferences: MD in medicine and SMD in social sciences. In ecology, almost half of all meta‐analyses use another outcome measure, the log‐transformed ratio of means (RoM), also called the response ratio.^2^, ^3^ We plan to discuss RoM in a separate paper.

If the studies can be assumed to have the same true effect, a meta‐analysis uses a fixed‐effect (FE) model (common‐effect model) to combine the estimates. Otherwise, the studies' true effects can depart from homogeneity in a variety of ways. Most commonly, a random‐effects (RE) model regards those effects as a sample from a distribution and summarizes their heterogeneity via its variance, usually denoted by *τ* ^2^. (Another approach, which we do not discuss further, allows the studies' true effects to differ without following a distribution.^4^) The between‐studies variance, *τ* ^2^, has a key role in estimates of the mean of the distribution of random effects; but it is also important as a quantitative indication of heterogeneity,^5^ especially because the interpretation of the popular *I* ^2^ measure^6^ is problematic.^7^, ^8^ In studying estimation for meta‐analysis of MD and SMD, we focus first on *τ* ^2^ and then proceed to the overall effect.

Veroniki et al^9^ provide a comprehensive overview and recommendations on general‐purpose methods (which can be used with any measure of effect) of estimating *τ* ^2^ and its uncertainty. Such a review, however, does not take into account the important evidence that the performance of those methods varies widely among effect measures. Veroniki et al^9^(Section 6.1) mention this variation only in passing, as a hypothetical possibility. To address this important issue, we introduce new methods, specific to MD and SMD, that could perform better than the general‐purpose ones.

Veroniki et al^9^ recommend four methods of estimating *τ* ^2^: the well‐established methods of DerSimonian and Laird,^10^ Mandel and Paule,^11^ and restricted maximum likelihood, and the less‐familiar method of Jackson.^12^ Three of these four methods match moments to the asymptotic distribution of Cochran's *Q* statistic, and the fourth ignores the randomness of the inverse‐variance weights. However, they all may be applicable only for large sample sizes.

As an alternative, we use improved effect‐measure‐specific approximations to the expected value of *Q* for both MD^13^ and SMD^14^ to introduce two new moment‐based point estimators of *τ* ^2^ for MD (Welch‐type [WT] and corrected DerSimonian‐Laird [CDL]) and one WT interval estimator. We also introduce one moment‐based point estimator and one interval estimator for *τ* ^2^ in SMD.

Any review on comparative performance of the existing methods, such as Veroniki et al,^9^ currently can draw on limited empirical information, which we summarize in Appendix A in the Supplementary Materials. Existing gaps in evidence for MD include a complete lack of simulations using unpooled estimators of the study‐level variance; instead, some studies have used the pooled estimator, and others, equivalently, have generated one normally distributed effect measure and an independent chi‐squared estimate of the variance. The pooled estimator is equivalent to the unpooled estimator only when the sample sizes are equal within the study. So far, the only two studies^12^, ^15^ of coverage investigated a very limited number of interval estimators of *τ* ^2^. In addition, studies have not examined the effect of estimation of *τ* ^2^ on coverage of the overall mean (Petropoulou and Mavridis^16^ consider only inverse‐variance‐weighted estimators). For SMD, no studies have investigated coverage of *τ* ^2^. Only one study^17^ investigated coverage of the overall SMD, *δ*, but only for *δ*=0.5.

Therefore, we undertook an extensive simulation study to evaluate our new methods of estimating heterogeneity variance for MD and SMD and to compare them with existing methods, aiming also to address the gaps in evidence. We also study coverage of confidence intervals for *τ* ^2^ achieved by five methods, comparing our Q‐profile methods based on improved approximations to the distribution of Cochran's *Q* with the Q‐profile method of Viechtbauer,^18^ profile‐likelihood‐based intervals, and methods by Biggerstaff and Jackson^19^ and Jackson.^12^

For each estimator of *τ* ^2^, we also study bias of the corresponding inverse‐variance‐weighted estimator of the overall effect. As our work progressed, it became clear that those inverse‐variance‐weighted estimators generally had unacceptable bias for SMD. Therefore, we added an estimator (SSW) whose weights depend only on the sample sizes of the treatment and control arms. We study the coverage of the confidence intervals associated with the inverse‐variance‐weighted estimators, and also the HKSJ interval, the HKSJ interval using the improved estimator of *τ* ^2^, and the interval centered at SSW and using the improved ${\hat{\tau }}^{2}$ in estimating its variance.

The structure of this paper is as follows. In Section 2, we briefly review the continuous effect measures MD and SMD. Section 3 describes the standard random‐effects model. Section 4 lists the methods for point estimation and interval estimation of a between‐study variance. Section 5 lists the methods for point and interval estimation of the overall effect. Section 6 reports on our extensive simulation study. Section 7 discusses an example for SMD. Section 8 concludes with a discussion of practical implications for meta‐analysis of MD and SMD, including recommendations on the choice of methods.

## 2 MD AND SMD

We assume that each of the *K* studies in the meta‐analysis consists of two arms, treatment(T) and control (C), with sample sizes *n*_*iT*_ and *n*_*iC*_. The total sample size in study *i* is *n*_*i*_=*n*_*iT*_+*n*_*iC*_. We denote the ratio of the control sample size to the total by *q*_*i*_=*n*_*iC*_/*n*_*i*_. The subject‐level data in each arm are assumed to be normally distributed with means *μ*_*iT*_ and *μ*_*iC*_ and variances $\sigma_{iT}^{2}$ and $\sigma_{iC}^{2}$. The sample means are ${\bar{x}}_{i\;j}$, and the sample variances are $s_{i\;j}^{2}$, for *i*=1,…,*K* and *j*=*C* or *T*.

### 2.1 Mean difference

The MD effect measure is

$$\mu_{i}=\mu_{iT}-\mu_{iC},\;\text{estimated by}\;y_{i}={\bar{x}}_{iT}-{\bar{x}}_{iC},$$

with variance $\sigma_{i}^{2}=\sigma_{iT}^{2}/n_{iT}+\sigma_{iC}^{2}/n_{iC}$, estimated by

(1) $$v_{i}^{2}={\hat{\sigma }}_{i}^{2}=s_{iT}^{2}/n_{iT}+s_{iC}^{2}/n_{iC}.$$

$s_{iT}^{2}$ and $s_{iC}^{2}$ do not depend on *μ*_*iT*_ and *μ*_*iC*_, so ${\hat{\sigma }}_{i}^{2}$ does not involve *μ*_*i*_. In the best‐case scenario for traditional meta‐analysis methods, for normal data, the sample means are independent of the sample variances (and therefore of inverse‐variance‐based weights). However, the relation of the between‐study variance *τ* ^2^ and the within‐study variances $\sigma_{i}^{2}$ may affect quality of estimation. Sometimes the pooled sample variance, given by Equation (2), is used instead of $v_{i}^{2}$. Then, however, unequal variances in the treatment and control arms can adversely affect estimation.^13^

### 2.2 Standardized mean difference

The SMD effect measure is

$$\delta_{i}=\frac{\mu_{iT}-\mu_{iC}}{\sigma_{i}}.$$

The variances in the treatment and control arms are usually assumed to be equal. Therefore, *σ*_*i*_ is estimated by the square root of the pooled sample variance

(2) $$s_{i}^{2}=\frac{(n_{iT}-1)s_{iT}^{2}+(n_{iC}-1)s_{iC}^{2}}{n_{iT}+n_{iC}-2}.$$

The plug‐in estimator $d_{i}=({\bar{x}}_{iT}-{\bar{x}}_{iC})/s_{i}$, known as Cohen's *d*, is biased in small samples, and we do not consider it further. Instead, we study the unbiased estimator

$$g_{i}=J(m_{i})\frac{{\bar{x}}_{iT}-{\bar{x}}_{iC}}{s_{i}},$$

where *m*_*i*_=*n*_*iT*_+*n*_*iC*_−2, and the factor

$$J(m)=\frac{\Gamma \left(\frac{m}{2}\right)}{\sqrt{\frac{m}{2}}\Gamma \left(\frac{m-1}{2}\right)},$$

often approximated by 1−3/(4 *m*−1), corrects for bias.^20^ This estimator of *δ* is sometimes called Hedges's *g*. For the variance of *g*_*i*_, we use the unbiased estimator

(3) $$v_{i}^{2}=\frac{n_{iT}+n_{iC}}{n_{iT}n_{iC}}+\left(1-\frac{\left(m_{i}-2\right)}{m_{i}J{\left(m_{i}\right)}^{2}}\right)g_{i}^{2},$$

derived by Hedges.^20^ The sample SMD *g*_*i*_ has a scaled noncentral *t*‐distribution with noncentrality parameter [*n*_*i*_ *q*_*i*_(1−*q*_*i*_)]^1/2^ *δ*_*i*_:

(4) $$\frac{\sqrt{n_{i}q_{i}(1-q_{i})}}{J(m_{i})}g_{i}∼t_{m_{i}}\left({\left[n_{i}q_{i}(1-q_{i})\right]}^{1/2}\delta_{i}\right).$$

Cohen^21^ categorized values of *δ*=0.2,0.5,0.8 as small, medium, and large effect sizes. However, these definitions of “small,” “medium,” and “large” may not be appropriate outside the behavioral sciences. Ferguson^22^ proposed the values 0.41,1.15,2.70 as benchmarks in the social sciences. In an empirical study of 21 ecological meta‐analyses by Møller and Jennions,^23^ 136 observed values of SMD varied in magnitude from 0.005 to 3.416, with mean 0.721 and 95% confidence interval (0.622‐0.820). Unfortunately, little is known about the range of *τ* ^2^ for SMD in various applications.

## 3 STANDARD RANDOM‐EFFECTS MODEL

In meta‐analysis, the standard random‐effects model assumes that within‐ and between‐study variabilities are accounted for by approximately normal distributions of within‐ and between‐study effects. For a generic measure of effect,

(5) $${\hat{\theta }}_{i}∼N\left(\theta_{i},\sigma_{i}^{2}\right)\;\text{and}\;\theta_{i}∼N(\theta ,\tau^{2}),$$

resulting in the marginal distribution ${\hat{\theta }}_{i}∼N(\theta ,\sigma_{i}^{2}+\tau^{2})$. ${\hat{\theta }}_{i}$ is the estimate of the effect in Study *i*, and its within‐study variance is $\sigma_{i}^{2}$, estimated by ${\hat{\sigma }}_{i}^{2}$, *i*=1,…,*K*. *τ* ^2^ is the between‐study variance, which is estimated by ${\hat{\tau }}^{2}$. The overall effect *θ* can be estimated by the weighted mean

(6) $${\hat{\theta }}_{\text{RE}}=\frac{\overset{K}{\underset{i=1}{\sum }}{ŵ}_{i}({\hat{\tau }}^{2}){\hat{\theta }}_{i}}{\overset{K}{\underset{i=1}{\sum }}{ŵ}_{i}({\hat{\tau }}^{2})},$$

where the ${ŵ}_{i}({\hat{\tau }}^{2})={\left({\hat{\sigma }}_{i}^{2}+{\hat{\tau }}^{2}\right)}^{-1}$ are inverse‐variance weights. The FE estimate $\hat{\theta }$ uses weights ${ŵ}_{i}={ŵ}_{i}(0)$.

If $w_{i}=1/\mathrm{Var}({\hat{\theta }}_{i})$, the variance of the weighted mean of the ${\hat{\theta }}_{i}$ is $1/\sum w_{i}$. Thus, many authors estimate the variance of ${\hat{\theta }}_{\text{RE}}$ by ${\left[\sum_{i=1}^{K}{ŵ}_{i}({\hat{\tau }}^{2})\right]}^{-1}$. In practice, however, this estimate may not be satisfactory.^24^, ^25^, ^26^

## 4 METHODS OF ESTIMATING BETWEEN‐STUDY VARIANCE

### 4.1 Point estimators

Our study includes the four methods recommended by Veroniki et al^9^: DerSimonian‐Laird (DL), restricted maximum‐likelihood (REML), Mandel‐Paule (MP), and Jackson (J). In the interest of transparency and reproducibility, we review the details of these methods in Web Appendix B1. In Sections 4.1.1 and 4.1.2, we introduce two new methods for MD and one new method for SMD.

#### 4.1.1 Point estimation of ***τ*** ^2^ for MD by the WT and CDL methods

Because the ${ŵ}_{i}({\hat{\tau }}^{2})$ in (6) involve the ${\hat{\sigma }}_{i}^{2}$, *K*−1 is an adequate approximation for the expected value of Cochran's *Q* statistic only for very large sample sizes. However, this approximation is used in all moment methods for estimating *τ* ^2^. As an alternative one can use an improved, effect‐measure‐specific approximation to the expected value of *Q*. Corrected MP‐type moment‐based methods for estimating *τ* ^2^ equate the *Q* statistic, with weights ${ŵ}_{i}(\tau^{2})$, to the first moment of an improved approximate null distribution of *Q*. Corrected DerSimonian‐Laird–type methods equate the *Q* statistic, with weights ŵ(0), to the first nonnull moment of *Q*.

More‐realistic approximations to the null distribution of *Q* are available for several effect measures. These approximations do not treat the estimates ${\hat{\sigma }}_{i}^{2}$ as equal to $\sigma_{i}^{2}$. For MD, Kulinskaya et al^13^ proposed an approximation based on the method of Welch.^27^ This method calculates corrected first two moments of *Q*, *κ*_1_=E[*Q*] and *κ*_2_=Var[*Q*], under the null hypothesis of homogeneity and then approximates the null distribution of *Q* by an F distribution: $ĉF_{K-1,{\hat{f}}_{2}}$ with matched moments. The estimated degrees of freedom ${\hat{f}}_{2}$ and the scale factor ĉ are functions of *K*, the *n*_*iT*_ and *n*_*iC*_, and the ${\hat{\sigma }}_{iT}^{2}$ and ${\hat{\sigma }}_{iC}^{2}$.

To simplify notation, with $w_{i}=1/\mathrm{Var}({\hat{\theta }}_{i})$, let $W=\sum w_{i}$, $W_{\left(k\right)}=\sum {w_{i}}^{k}$, and *p*_*i*_=1−*w*_*i*_/*W*, and let

(7) $$\gamma_{i}=\left(\frac{\sigma_{iT}^{4}}{n_{iT}^{2}f_{iT}}+\frac{\sigma_{iC}^{4}}{n_{iC}^{2}f_{iC}}\right),$$

where *f*_*i* *j*_=*n*_*i* *j*_−1 is the number of degrees of freedom for group *j* of study *i*, *j*=*T*,*C*. Then, the null moments of *Q* for MD^13^ are

(8) $$\kappa_{1}\approx K-1+2\underset{i}{\sum }w_{i}^{2}\gamma_{i}\;p_{i}^{2};\;\kappa_{2}\approx 2(K-1)+14\underset{i}{\sum }w_{i}^{2}\gamma_{i}\;p_{i}^{2}.$$

We propose a new moment‐based estimator of *τ* ^2^ for MD based on this improved approximation. Let *E*_*WT*_(*Q*)=*κ*_1_ denote the corrected expected value of *Q*. Then, one obtains the WT estimator of *τ* ^2^ in the spirit of Mandel and Paule^11^ by substituting ${\hat{\sigma }}_{i}^{2}$ for $\sigma_{i}^{2}$ where $\mathrm{Var}({\hat{\theta }}_{i})$ appears in *κ*_1_ to obtain ${\hat{E}}_{WT}(Q)$ and iteratively solving

(9) $$Q(\tau^{2})=\overset{K}{\underset{i=1}{\sum }}\frac{{\left(\theta_{i}-{\hat{\theta }}_{RE}\right)}^{2}}{{\hat{\sigma }}_{i}^{2}+\tau^{2}}={\hat{E}}_{WT}(Q).$$

We denote the resulting estimator of *τ* ^2^ by ${\hat{\tau }}_{WT}^{2}$. This proposal assumes that using the true *τ* ^2^ in the denominator in *Q*(*τ* ^2^) would achieve the null value of *E*(*Q*). However, this assumption is motivated by the standard assumption of a chi‐squared distribution and, as we show in Section 6.1.2, is disproved by simulations. This is not surprising, as the null distribution of Q is better approximated by an F distribution.^13^

We also propose another new moment‐based estimator of *τ* ^2^ for MD based on the improved first moment of *Q* and the same term in *τ* ^2^ as in DerSimonian‐Laird.^10^ With

$$E(Q)\approx K-1+2\underset{i}{\sum }w_{i}^{2}\gamma_{i}p_{i}^{2}+\tau^{2}(W-W_{\left(2\right)}/W),$$

and substituting ${\hat{\sigma }}_{i}^{2}$ for $\sigma_{i}^{2}$ in E(*Q*) (as above) and *Q* for E(*Q*), the CDL estimator is given by

$${\hat{\tau }}_{CDL}^{2}=\max\left(\frac{Q-(K-1)-2{\text{∑}}_{i}\;{ŵ}_{i}^{2}{\hat{\gamma }}_{i}{\hat{p}}_{i}^{2}}{Ŵ-{Ŵ}_{\left(2\right)}/Ŵ},0\right).$$

The difference from the WT estimator is that CDL uses the improved nonnull first moment of *Q*.

#### 4.1.2 Point estimation of ***τ*** ^2^ for SMD by the Kulinskaya‐Dollinger‐Bjørkestøl method

For SMD, Kulinskaya et al^14^ derived *O*(1/*n*) corrections to moments of *Q* and suggested using the chi‐squared distribution with degrees of freedom equal to the estimate of the corrected first moment to approximate the distribution of *Q*. Kulinskaya et al^14^ give expressions from which it can be calculated, along with a computer program in R.

We propose a new moment‐based estimator of *τ* ^2^ for SMD in the spirit of Mandel and Paule^11^ based on this improved approximation. Let *E*_*KDB*_(*Q*) denote the corrected expected value of *Q*. Then, one obtains the KDB estimate of *τ* ^2^ by iteratively solving Equation (9) with *E*_*KDB*_(*Q*) instead of *E*_*WT*_(*Q*) in the right‐hand side.

We denote the resulting estimator of *τ* ^2^ by ${\hat{\tau }}_{KDB}^{2}$.

### 4.2 Interval estimators

Among the confidence‐interval methods reviewed by Veroniki et al,^9^ our study includes four: profile‐likelihood (PL), Q‐profile (QP), Biggerstaff and Jackson (BJ), and Jackson (J). (Veroniki et al consider combinations of a point estimator and an interval estimator, and they point out that some combinations are not appropriate, because the interval estimator may yield CIs that do not contain the particular point estimate of the between‐studies variance.) We review the details of these methods in Web Appendix B2. In Section 4.2.1, we introduce two new interval estimators, one for MD and the other for SMD.

#### 4.2.1 WT interval and Kulinskaya‐Dollinger‐Bjørkestøl interval

We propose a new WT confidence interval for the between‐study variance for MD. This interval for *τ* ^2^ combines the Q‐profile approach and the improved approximation by Kulinskaya et al^13^ based on the method of Welch^27^ (ie, the scaled F distribution with *K*−1 and ${\hat{f}}_{2}$ degrees of freedom based on the corrected first two moments of *Q*).

This corrected Q‐profile confidence interval can be estimated from the lower and upper quantiles of *F*_*Q*_, the cumulative distribution function for the improved approximation to the distribution of *Q*:

(10) $$Q(\tau_{L}^{2})=F_{Q;0.975}\;Q(\tau_{U}^{2})=F_{Q;0.025}$$

The upper and lower confidence limits for *τ* ^2^ can be calculated iteratively.

Similarly, when the effect measure is SMD, the Kulinskaya‐Dollinger‐Bjørkestøl (KDB) confidence interval for *τ* ^2^ is based on the chi‐squared distribution with the corrected first moment developed by Kulinskaya et al.^14^

## 5 METHODS OF ESTIMATING OVERALL EFFECT

Most of the point estimators of the overall effect have corresponding interval estimators, but some do not. Therefore, we describe point estimators and interval estimators in separate sections.

### 5.1 Point estimators

A random‐effects method that estimates *θ* by a weighted mean with inverse‐variance weights, as in Equation (6), is determined by the particular ${\hat{\tau }}^{2}$ that it uses in ${ŵ}_{i}({\hat{\tau }}^{2})$. The best‐known and most widely used estimator, ${\hat{\theta }}_{\text{DL}}$, was introduced by DerSimonian and Laird^10^; it uses ${\hat{\tau }}_{\text{DL}}^{2}$. Its shortcomings, in particular bias and below‐nominal coverage of the companion confidence interval, have led numerous authors to propose alternative estimators of *τ* ^2^. Some of those shortcomings arose from the derivation underlying ${\hat{\tau }}_{\text{DL}}^{2}$, which uses the $\sigma_{i}^{2}$ and *τ* ^2^ and then substitutes the ${\hat{\sigma }}_{i}^{2}$ and ${\hat{\tau }}^{2}$. Unfortunately, the alternative methods REML, J, and MP generally rely on that same unsupported substitution; for MD, CDL attempts to reduce its impact.

In an attempt to reduce the bias in estimating the overall SMD that we encountered in the inverse‐variance‐weighted estimators, we included a point estimator whose weights depend only on the studies' sample sizes.^28^, ^29^ For this estimator (SSW), $w_{i}={\tilde{n}}_{i}=n_{iT}n_{iC}/(n_{iT}+n_{iC})$; that is, *w*_*i*_ omits the term in $g_{i}^{2}$ in Equation (3); ${\tilde{n}}_{i}$ is the effective sample size in Study *i*.

### 5.2 Interval estimators

The point estimators DL, REML, J, MP, WT, CDL, and KDB have companion interval estimators of *θ*. The customary approach estimates the variance of ${\hat{\theta }}_{\text{RE}}$ by ${\left[\sum_{i=1}^{K}{ŵ}_{i}({\hat{\tau }}^{2})\right]}^{-1}$ and bases the half‐width of the interval on the normal distribution. That expression for the variance of ${\hat{\theta }}_{\text{RE}}$ would be correct if it were based on $w_{i}={\left(\sigma_{i}^{2}+\tau^{2}\right)}^{-1}$. In practice, however, using ${ŵ}_{i}({\hat{\tau }}^{2})$ may not yield a satisfactory approximation. In addition, we have not seen empirical evidence that the sampling distributions of ${\hat{\theta }}_{\text{RE}}$ for the various choices of estimator for *τ* ^2^ are adequately approximated by a normal distribution.

Hartung and Knapp^30^ and, independently, Sidik and Jonkman^31^ developed an estimator for the variance of ${\hat{\theta }}_{\text{RE}}$ that takes into account the variability of the ${\hat{\sigma }}_{i}^{2}$ and ${\hat{\tau }}^{2}$. The Hartung‐Knapp‐Sidik‐Jonkman (HKSJ) confidence interval uses the estimator

(11) $${\hat{\mathrm{Var}}}_{\text{HKSJ}}({\hat{\theta }}_{\text{DL}})=\overset{K}{\underset{i=1}{\sum }}{ŵ}_{i}\left({\hat{\tau }}_{\text{DL}}^{2}\right){\left({\hat{\theta }}_{i}-{\hat{\theta }}_{DL}\right)}^{2}/\left[(K-1)\overset{K}{\underset{i=1}{\sum }}{ŵ}_{i}\left({\hat{\tau }}_{\text{DL}}^{2}\right)\right],$$

together with critical values from the *t* distribution on *K*−1 degrees of freedom. A potential weakness is that the derivation of the variance estimator and the *t* distribution uses the $\sigma_{i}^{2}$ and *τ* ^2^ and then substitutes the ${\hat{\sigma }}_{i}^{2}$ and ${\hat{\tau }}_{DL}^{2}$. In addition, the HKSJ interval uses ${\hat{\theta }}_{\text{DL}}$ as its midpoint, so it will have any bias that is present in ${\hat{\theta }}_{\text{DL}}$. We study a modification of HKSJ that uses the WT estimator or KDB estimator of *τ* ^2^ and uses ${\hat{\theta }}_{\text{WT}}$ or ${\hat{\theta }}_{\text{KDB}}$, respectively, as the midpoint.

The interval estimators corresponding to SSW (SSW WT, SSW CDL, and SSW KDB) use the SSW point estimator as the midpoint, and the half‐width equals the estimated standard deviation of SSW under the random‐effects model times the critical value from the *t* distribution on *K*−1 degrees of freedom. The estimator of the variance of SSW is

(12) $$\hat{\mathrm{Var}}({\hat{\theta }}_{\text{SSW}})=\frac{\sum {\tilde{n}}_{i}^{2}\left(v_{i}^{2}+{\hat{\tau }}^{2}\right)}{{\left(\sum {\tilde{n}}_{i}\right)}^{2}},$$

in which $v_{i}^{2}$ comes from Equation (1) or (3) and ${\hat{\tau }}^{2}={\hat{\tau }}_{\text{WT}}^{2}$, ${\hat{\tau }}^{2}={\hat{\tau }}_{\text{CDL}}^{2}$, and ${\hat{\tau }}^{2}={\hat{\tau }}_{\text{KDB}}^{2}$, respectively.

## 6 SIMULATION STUDY

As mentioned in Section 1, other studies have used simulation to examine estimators of *τ* ^2^ or of the overall effect for MD or SMD, but gaps in evidence remain. Appendix A in the Supplementary Materials contains a detailed summary of previous simulation studies and provides our rationale for choosing the ranges of values for *μ*, *δ*, and *τ* ^2^ that we consider realistic for a range of applications.

The following paragraphs describe mainly features that are common to our simulations for MD and SMD. Sections 6.1.1 and 6.2.1 describe other features that are specific to those measures.

All simulations use the same numbers of studies *K*=5,10,30 and, for each combination of parameters, the same vector of total sample sizes *n*=(*n*_1_,…,*n*_*K*_) and the same proportions of observations in the control arm *q*_*i*_=.5,.75 for all *i*. The sample sizes in the treatment and control arms are *n*_*iT*_=⌈(1−*q*_*i*_)*n*_*i*_⌉ and *n*_*iC*_=*n*_*i*_−*n*_*iT*_, *i*=1,…,*K*. The values of *q* reflect two situations for the two arms of each study: approximately equal (1:1) and quite unbalanced (1:3).

We study equal and unequal study sizes. For equal study sizes, *n*_*i*_ is as small as 20, and for unequal study sizes, *n*_*i*_ is as small as 12, in order to examine how the methods perform for the extremely small sample sizes that arise in some areas of application.

In choosing unequal study sizes, we follow a suggestion of Sánchez‐Meca and Marín‐Martínez,^32^ who selected study sizes having skewness of 1.464, which they considered typical in behavioral and health sciences. Tables 1 and 2 give the details.

**Table 1:** Data patterns in the simulations for mean difference (MD)

MD	Equal study sizes	Unequal study sizes	Full results in
			Bakbergenuly et al^33^
			Appendices:
*K* (number of studies)	5, 10, 30	5, 10, 30	
*n* or $\bar{n}$ (average (individual) study size—total of the two arms)	20, 40, 100, 250	30 (12, 16, 18, 20, 84),	
For *K*=10 and *K*=30, the same set of unequal study sizes		60 (24, 32, 36, 40, 168),	
is used twice or six times, respectively		100 (64, 72, 76, 80, 208),	
		160 (124, 132, 136, 140, 268)	
*q* (proportion of each study in the control arm)	1/2, 3/4	1/2, 3/4	
First series of within‐study variances:			
*μ*	0	0	B1, B2; B3, B4
$\sigma_{C}^{2},\sigma_{T}^{2}$(within‐study variances)	(1,1), (1,2)	(1,1), (1,2)	
*τ* ^2^ (variance of random effect)	0(0.01)0.1(0.1)1	0(0.01)0.1(0.1)1	A1, A2; A3, A4
Second series of within‐study variances:			
*μ*	0	0	B5, B6
$\sigma_{C}^{2},\sigma_{T}^{2}$ (within‐study variances)	(10,10), (10,20)	(10,10), (10,20)	
*τ* ^2^ (variance of random effect)	0(0.1)1	0(0.1)1	A5, A6

**Table 2:** Data patterns in the simulations for standardized mean difference (SMD)

SMD	Equal study sizes	Unequal study sizes	Full results in
			Bakbergenuly et al^34^
			Appendices:
*K* (number of studies)	5, 10, 30	5, 10, 30	
*n* or $\bar{n}$ (average (individual) study size—total of the two arms)	20, 40, 100, 250	30 (12, 16, 18, 20, 84),	
	30, 50, 60, 70	60 (24, 32, 36, 40, 168),	
For *K*=10 and *K*=30, the same set of unequal study sizes		100 (64, 72, 76, 80, 208),	
is used twice or six times, respectively		160 (124, 132, 136, 140, 268)	
*q* (proportion of each study in the control arm)	1/2, 3/4	1/2, 3/4	
*δ* (true value of the SMD)	0, 0.2, 0.5, 1, 2	0, 0.2, 0.5, 1, 2	B1, B2
*τ* ^2^ (variance of random effect)	0(0.5)2.5	0(0.5)2.5	A1, A2

The patterns of sample sizes are illustrative; they do not attempt to represent all patterns seen in practice. By using the same patterns of sample sizes for each combination of the other parameters, we avoid the additional variability in the results that would arise from choosing sample sizes at random (eg, uniformly between 20 and 200).

We use a total of 10 000 repetitions for each combination of parameters. Thus, the simulation standard error for estimated coverage of *τ* ^2^, *μ*, or *δ* at the 95% confidence level is roughly $\sqrt{0.95\times 0.05/10,000}=0.00218$.

The simulations were programmed in R version 3.3.2 using the University of East Anglia 140‐computer‐node High Performance Computing (HPC) Cluster, providing a total of 2560 CPU cores, including parallel processing and large memory resources. For each configuration, we divided the 10 000 replications into 10 parallel sets of 1000 replications.

The structure of the simulations invites an analysis of the results along the lines of a designed experiment, in which the variables are *τ* ^2^, *n*, *K*, *q*, $\sigma_{C}^{2}$, and $\sigma_{T}^{2}$. Most of the variables are crossed, but two have additional structure. Within the two levels of *n*, equal and unequal, the values are nested: *n*=20,40,100,250 and $\bar{n}=30,\;60,\;100,\;160$. The values of $\sigma_{C}^{2}$ and $\sigma_{T}^{2}$ consist of a cross of two factors, equal/unequal and small/large ( $\sigma_{C}^{2}=1$ and $\sigma_{T}^{2}=1$, $\sigma_{C}^{2}=10$ and $\sigma_{T}^{2}=10$, $\sigma_{C}^{2}=1$ and $\sigma_{T}^{2}=2$, and $\sigma_{C}^{2}=10$ and $\sigma_{T}^{2}=20$). We approach the analysis of the data from the simulations qualitatively, to identify the variables that substantially affect (or do not affect) the performance of the estimators as a whole and the variables that reveal important differences in performance. We might hope to describe the estimators' performance one variable at a time, but such “main effects” often do not provide an adequate summary: important differences are related to certain combinations of two or more variables.

We use this approach to examine bias and coverage in estimation of *τ* ^2^ and bias and coverage in estimation of *μ* and *δ*. Our summaries of results include illustrative figures and are based on examination of the figures in the corresponding arXiv e‐prints.^33^, ^34^ Sections 6.1.2 and 6.2.2 give brief summaries, and Appendices D and E in the Supplementary Materials contain more details.

A reviewer inquired about the values of *I* ^2^ underlying our simulations. Figures C1 and C2 in Appendix C plot *I* ^2^=100*τ* ^2^/(*τ* ^2^+*s* ^2^) versus *τ* ^2^∈[0,1] for MD and versus *δ* for SMD when *τ* ^2^=0.5(0.5)2, with traces for *n* = 20, 40, 100, 250. As indicated by the definition, *I* ^2^ increases as *τ* ^2^ increases. The value of *n* also has a substantial impact (larger *n* yields higher *I* ^2^); Higgins and Thompson^6^ did not construct *I* ^2^ to be independent of the precisions of estimates observed in the studies. Importantly, for SMD, *I* ^2^ decreases as *δ* increases, especially for the smaller *n*, contrary to the scale‐invariance criterion of Higgins and Thompson. We emphasize that we discourage use of *I* ^2^, for the reasons mentioned here and in Section 1.

### 6.1 Mean difference

#### 6.1.1 Design

For the MD, we vary six parameters: the between‐study variance (*τ* ^2^) and the within‐study variances ( $\sigma_{T}^{2}$ and $\sigma_{C}^{2}$), in addition to the number of studies (*K*), the total sample size (*n* and $\bar{n}$), and the proportion of observations in the control arm (*q*). Table 1 lists the values of each parameter. We set the overall true MD *μ*=0 because the estimators of *τ* ^2^ do not involve *μ* and the estimators of *μ* are equivariant.

To cover both small and large values of the ratio of within‐study to between‐studies variance, separately from the value of *τ* ^2^, we use two series of within‐study variances ( $\sigma_{C}^{2},\sigma_{T}^{2}$=(1,1), (1,2) and $\sigma_{C}^{2},\sigma_{T}^{2}$=(10,10), (10,20)). We generate the within‐study sample variances $s_{i\;j}^{2}$ ( *j*=*T*,*C*) from chi‐squared distributions as $\sigma_{i\;j}^{2}\chi_{n_{i\;j}-1}^{2}/(n_{ij}-1)$. We generate the estimated MDs *y*_*i*_ from a normal distribution with mean *μ* and variance $\sigma_{iT}^{2}/n_{iT}+\sigma_{iC}^{2}/n_{iC}+\tau^{2}$. We obtain the estimated within‐study variances as $v_{i}^{2}=s_{iT}^{2}/n_{iT}+s_{iC}^{2}/n_{iC}$.

The simulation standard error in the estimates of *μ* is 0.01 (for *n*=20) or less for the first series of within‐study variances, and 0.02 or less for the second series.

We study six point estimators of *τ* ^2^ (DL, REML, MP, J, WT, and CDL), five interval estimators of *τ* ^2^ (PL, QP, BJ, J, and WT), and ten interval estimators of *μ* (DL, REML, MP, J, WT, CDL, HKSJ, HKSJ WT, SSW WT, and SSW CDL).

#### 6.1.2 Results

**Figure 1:**
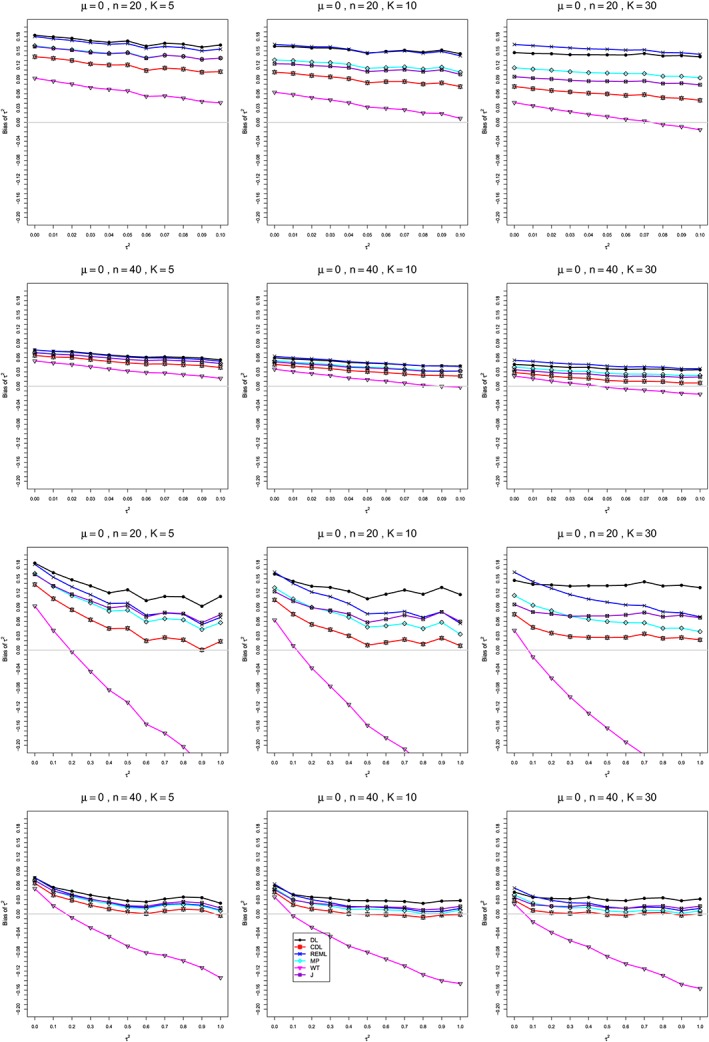
Mean difference: Bias of estimators of between‐studies variance *τ* ^2^∈[0,0.1] (top two rows) and *τ* ^2^∈[0,1] (bottom two rows) for *μ*=0, *q*=0.75 when $\sigma_{C}^{2}=1$, $\sigma_{T}^{2}=2$, *n*=20,40, and *K*=5,10,30. Light gray line at 0. CDL, corrected DL; DL, DerSimonian‐Laird; J, Jackson; MP, Mandel‐Paule; REML, restricted maximum‐likelihood; WT, Welch‐type [Colour figure can be viewed at http://wileyonlinelibrary.com]

**Figure 2:**
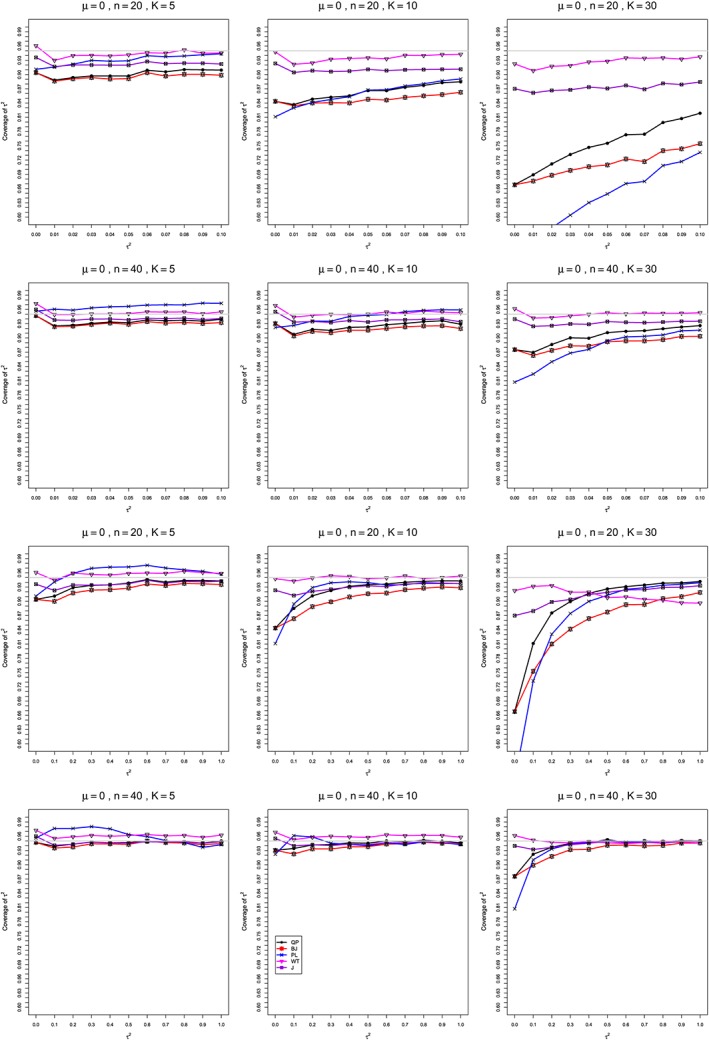
Mean difference: Coverage at the nominal 95% level of interval estimators of between‐studies variance *τ* ^2^∈[0,0.1] (top two rows) and *τ* ^2^∈[0,1] (bottom two rows) for *μ*=0, *q*=0.75, when $\sigma_{C}^{2}=1$, $\sigma_{T}^{2}=2$, *n*=20,40, and *K*=5,10,30. Light gray line at 0.95. BJ, Biggerstaff and Jackson; J, Jackson; PL, profile‐likelihood; QP, Q‐profile; WT, Welch‐type [Colour figure can be viewed at http://wileyonlinelibrary.com]

**Figure 3:**
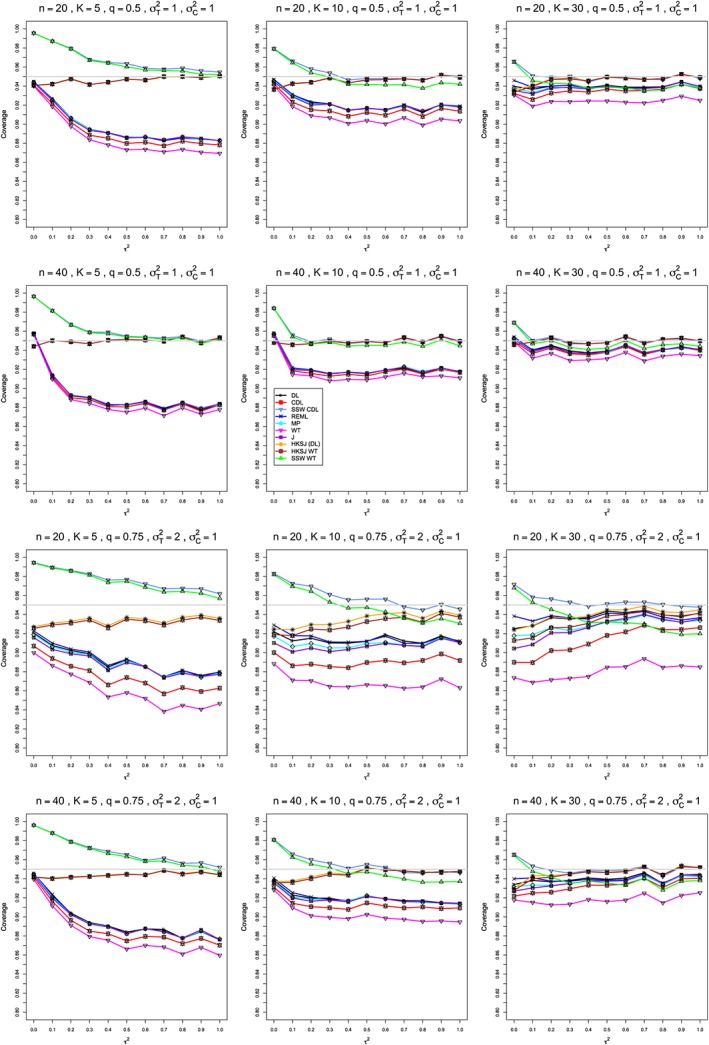
Mean difference: Coverage of 95*%* confidence intervals for *μ*. The between‐studies variance *τ* ^2^∈[0,1]. In the top two rows, *q*=.5, $\sigma_{C}^{2}=1$, and $\sigma_{T}^{2}=1$. In the bottom two rows, *q*=.75, $\sigma_{C}^{2}=1$, and $\sigma_{T}^{2}=2$. Light gray line at 0.95. CDL, corrected DL; DL, DerSimonian‐Laird; HKSJ, Hartung‐Knapp‐Sidik‐Jonkman; J, Jackson; MP, Mandel‐Paule; REML, restricted maximum‐likelihood; WT, Welch‐type [Colour figure can be viewed at http://wileyonlinelibrary.com]

Our full simulation results are available as an arXiv e‐print (Bakbergenuly et al^33^). They comprise 108 figures, each presenting a plot of bias, mean squared error (MSE) or coverage versus *τ* ^2^ for the four values of *n* or $\bar{n}$ and the three values of *K*. A short summary is given below and illustrated by Figures 1, 2, 3. A detailed description appears in Appendix D in the Supplementary Materials. Table 3 summarizes our recommendations.

**Table 3:** A summary of recommendations for meta‐analysis of mean difference (MD) and standardized MD (SMD)

Meta‐analysis of MD		
Estimation	*n*<**100**	*n* ≥ **100**
*τ* ^2^ point	All estimators are positively biased for small *n*	any estimator
	CDL is the least biased	
*τ* ^2^ interval	WT	any estimator other than PL
*μ* point	any estimator	any estimator
*μ* interval	HKSJ for balanced studies with *τ* ^2^<0.1 and *K* ≤ 10,	HKSJ
	where SSW CDL provides conservative coverage.	
	SSW CDL for unbalanced studies, or when *K* ≥ 20 and *τ* ^2^>0.1.	
**Meta‐analysis of SMD**		
*τ* ^2^ point	MP (somewhat underestimates) for *K* ≤ 10,	MP, KDB, or REML
	KDB (somewhat overestimates) for *K*>10	
*τ* ^2^ interval	QP	QP, PL, KDB
*δ* point	SSW, all other estimators have negative bias	any estimator
*δ* interval	HKSJ or HKSJ KDB for *δ*<0.5, SSW KDB for *δ* ≥ 0.5	HKSJ or HKSJ KDB or SSW KDB

Abbreviations: CDL, corrected DerSimonian‐Laird; HKSJ, Hartung‐Knapp‐Sidik‐Jonkman; KDB, Kulinskaya‐Dollinger‐Bjørkestøl; MP, Mandel‐Paule; PL, profile‐likelihood; QP, Q‐profile; REML, restricted maximum‐likelihood; WT, Welch‐type.

**Bias in estimation of** ***τ*** ^**2**^ **(Figure** 1 **)**

In summary, except for CDL and WT, the estimators of *τ* ^2^ (DL, REML, J, and MP) have nonnegligible positive bias, especially for small sample sizes (*n* ≤ 40) and small values of *τ* ^2^. Overall, CDL has the least bias, except for the most extreme cases, and is recommended for use in practice. WT is increasingly negatively biased for moderate to large heterogeneity, even for large sample sizes, so it is not recommended. All other estimators become acceptable for larger sample sizes (*n* ≥ 100).

**Coverage in estimation of** ***τ*** ^**2**^ **(Figure** 2 **)**

In summary, none of the interval estimators of *τ* ^2^ (PL, QP, BJ, J, and WT) consistently achieve coverage close to .95 (ie, between .94 and .96). All have difficulty at *τ* ^2^=0, usually overcoverage; the departures of PL extend to other small *τ* ^2^, and its coverage is often greater than .96 but sometimes less than .94. Meta‐analyses in which the studies have small sample sizes are challenging for PL, QP, BJ, and J, which in some situations have coverage well below nominal for all *τ* ^2^∈[0,1], especially when the number of studies is larger (*K*=30 vs. *K*=5 and *K*=10). Overall, WT comes closest to providing nominal coverage of *τ* ^2^. (The contrast in behavior between the WT interval and point estimators is surprising, but the former uses the appropriate F approximation to the distribution of *Q*, whereas the latter does not.)

**Bias in estimation of** ***μ***

Because the estimated MD and its estimated variance are independent, all the estimators of *μ* are practically unbiased in all situations.

**Coverage in estimation of** ***μ*** **(Figure** 3 **)**

When within‐study sample sizes are balanced, HKSJ and HKSJ WT generally (but not uniformly) have the best coverage for small *τ* ^2^ and *K*. Their coverage is not always within ±.01 of .95; it may be considerably below nominal for *τ* ^2^<0.1 when sample sizes are small, whereas SSW CDL provides conservative coverage; in situations where clear differences separate the interval estimators, HKSJ and HKSJ WT are much closer to .95. DL, WT, MP, REML, and J exhibit very serious undercoverage when *K*=5 and nontrivial undercoverage when *K*=10. For small and/or unbalanced sample sizes, SSW CDL is the only estimator achieving nominal coverage for larger values of *τ* ^2^ or *K*.

### 6.2 Standardized mean difference

#### 6.2.1 Design

For the SMD, we vary five parameters: the overall true SMD (*δ*) and the between‐studies variance (*τ* ^2^), in addition to the number of studies (*K*), the studies' total sample size (*n* and $\bar{n}$), and the proportion of observations in the control arm (*q*). Table 2 lists the values of each parameter.

We generate the true effect sizes *δ*_*i*_ from a normal distribution: *δ*_*i*_∼*N*(*δ*,*τ* ^2^). We generate the values of Hedges's estimator *g*_*i*_ directly from the appropriately scaled noncentral *t*‐distribution, given by Equation (4), and obtain their estimated within‐study variances from Equation (3).

We study five point estimators of *τ* ^2^ (DL, REML, MP, J, and KDB), five interval estimators of *τ* ^2^ (PL, QP, BJ, J, and KDB), six point estimators of *δ* (DL, REML, MP, J, KDB, and SSW), and eight interval estimators of *δ* (DL, REML, MP, J, KDB, HKSJ, HKSJ KDB, and SSW KDB).

#### 6.2.2 Results

**Figure 4:**
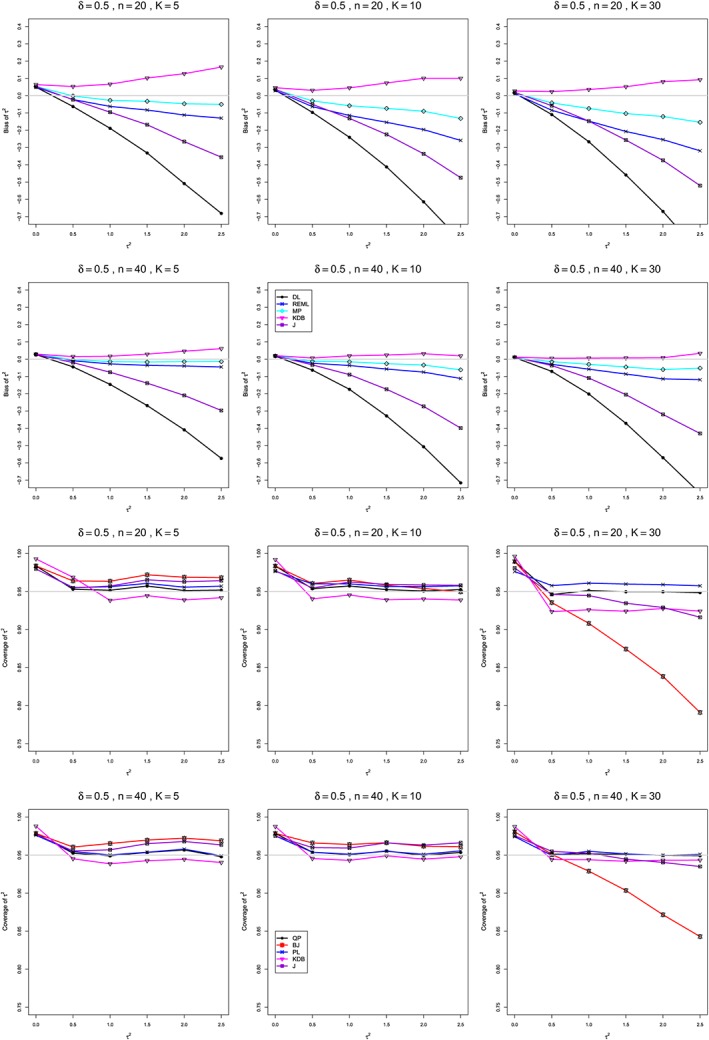
Standardized mean difference: Bias and coverage at nominal 95% level in estimation of between‐studies variance *τ* ^2^ for *δ*=0.5, *q*=.5, *n*=20,40, and *K*=5,10,30. Light gray line at 0 for bias and at 0.95 for coverage. BJ, Biggerstaff and Jackson; DL, DerSimonian‐Laird; KDB, Kulinskaya‐Dollinger‐Bjørkestøl; J, Jackson; MP, Mandel‐Paule; PL, profile‐likelihood; QP, Q‐profile; REML, restricted maximum‐likelihood [Colour figure can be viewed at http://wileyonlinelibrary.com]

**Figure 5:**
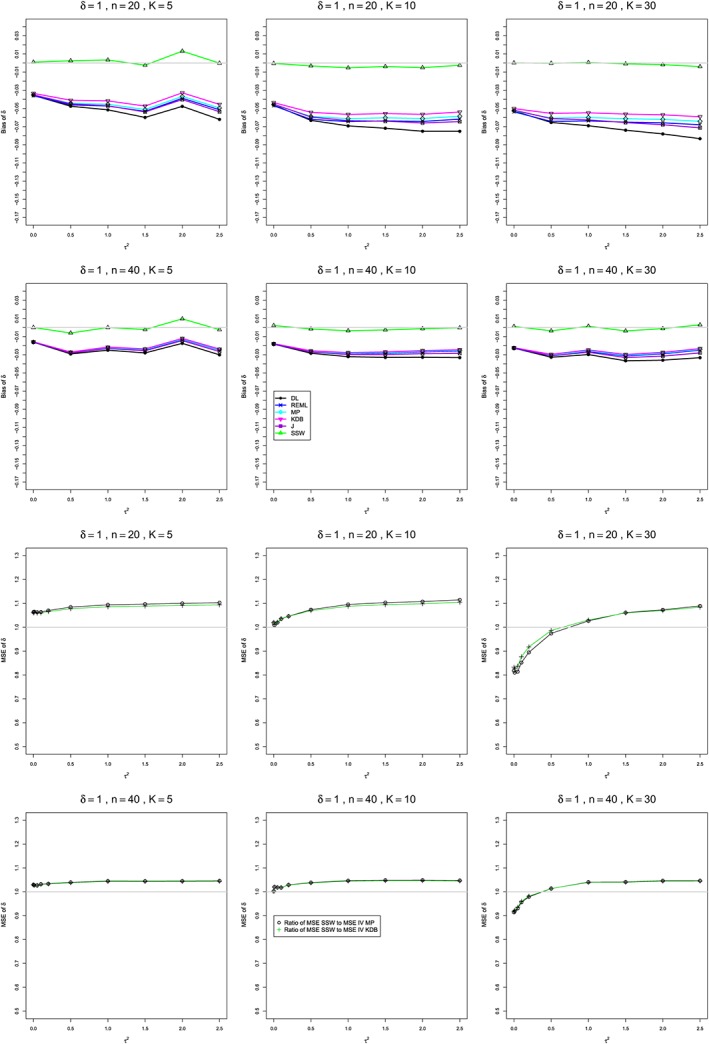
Standardized mean difference: Bias of the estimators of *δ* and ratio of mean squared errors (MSEs) of SSW to inverse‐variance‐weighted estimators when *δ*=1, *q*=.5, *n*=20,40, and *K*=5,10,30. Light gray line at 0 for bias and at 1 for the ratio of MSEs. DL, DerSimonian‐Laird; KDB, Kulinskaya‐Dollinger‐Bjørkestøl; J, Jackson; MP, Mandel‐Paule; REML, restricted maximum‐likelihood [Colour figure can be viewed at http://wileyonlinelibrary.com]

**Figure 6:**
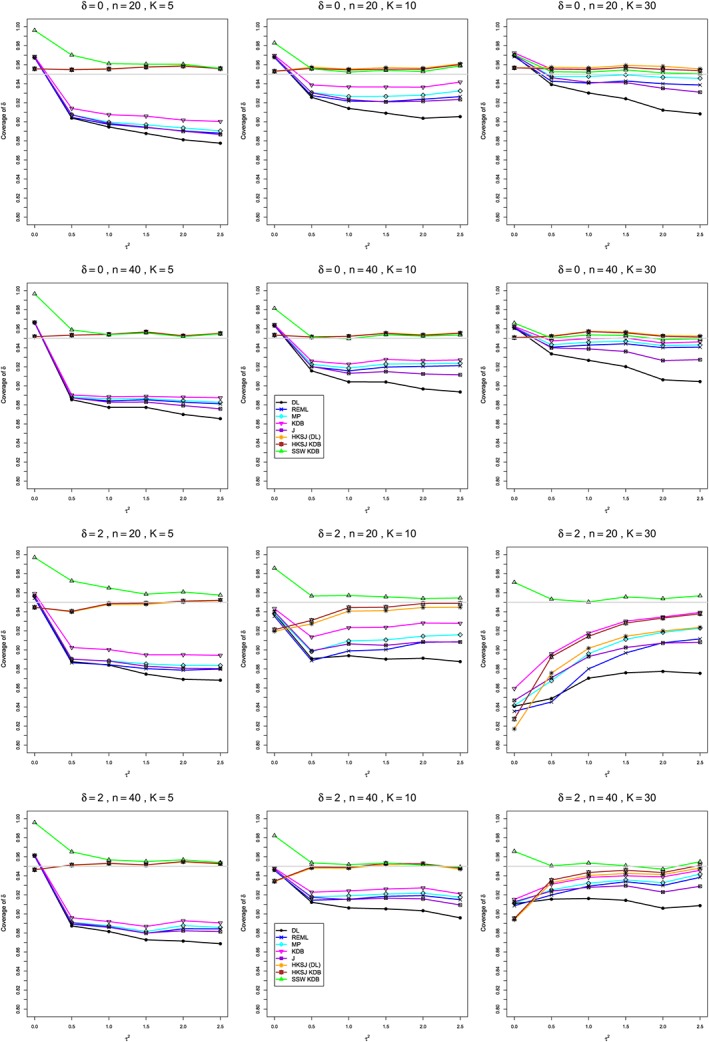
Standardized mean difference: Coverage of 95*%* confidence intervals for *δ* when *δ*=0 (top two rows) and 2 (bottom two rows), *q*=.5, *n*=20,40, and *K*=5,10,30. Light gray line at 0.95. DL, DerSimonian‐Laird; HKSJ, Hartung‐Knapp‐Sidik‐Jonkman; J, Jackson; KDB, Kulinskaya‐Dollinger‐Bjørkestøl; MP, Mandel‐Paule; REML, restricted maximum‐likelihood [Colour figure can be viewed at http://wileyonlinelibrary.com]

Our full simulation results are available as an arXiv e‐print (Bakbergenuly et al^34^). They comprise 130 figures, each presenting a plot of bias, MSE or coverage versus *τ* ^2^ for the four values of *n* or $\bar{n}$ and the three values of *K*. A short summary is given below and illustrated by Figures 4, 5, 6. In addition, Appendix H in Supplementary Materials has plots for *K*=20, *n*=20 and 40, and *δ*=0,0.5,1, and 2. A detailed description appears in Appendix E in the Supplementary Materials, and Table 3 summarizes our recommendations.

**Bias in estimation of** ***τ*** ^**2**^ **(Figure** 4 **)**

As Figure 4 (top) illustrates, the patterns of bias indicate a choice among the five estimators of *τ* ^2^ (DL, REML, J, MP, and KDB). When *n* ≤ 40, MP is closer to unbiased than KDB when *K*=5, the magnitudes of their biases are roughly equal when *K*=10, and KDB is closer to unbiased when *K*=30 (and also when *K*=20; see Appendix H). When *n* ≥ 100, MP, KDB, and REML are nearly unbiased. DL and J seriously underestimate *τ* ^2^. The average of MP and KDB should be close to unbiased.

**Coverage in estimation of** ***τ*** ^**2**^ **(Figure** 4 **)**

As Figure 4 (bottom) illustrates, all five interval estimators of *τ* ^2^ (PL, QP, BJ, J, and KDB) have coverage substantially above .95 when *τ* ^2^=0. When *τ* ^2^ ≥ 0.5, QP is generally closest to .95, whereas KDB is somewhat too liberal when *n*=20. The unusual behavior of BJ (and, to a lesser extent, J) when *K*=30 (and also when *K*=20; see Appendix H) adds to the evidence against it.

**Bias and MSE in estimation of** ***δ*** **(Figure** 5 **)**

The bias of SSW is close to 0, and the other five estimators (DL, REML, J, MP, and KDB), which use inverse‐variance weights, have greater (and negative) bias, amounting to 5% to 10% when sample sizes are small and *δ* ≥ 1. This bias increases as *τ* ^2^ and/or *δ* increases (see also Appendix H). SSW usually has slightly greater mean squared error than KDB and MP when *n* is small, but its MSE can be substantially smaller, especially for small *τ* ^2^.

**Coverage in estimation of** ***δ*** **(Figure** 6 **)**

Except when *δ* ≥ 1 and *K* ≥ 20 (see also Appendix H), HKSJ and HKSJ KDB have coverage closest to .95, though somewhat liberal; they differ little, and departures from .95 (toward lower coverage) are seldom serious. SSW KDB is rather conservative when *K*=5 and for other *K* when *τ* ^2^=0. Otherwise it provides reliable albeit slightly conservative coverage. When *δ*=2 and *K*=20 or 30, SSW KDB is substantially the best choice. All the estimators that use inverse‐variance weights and critical values from the normal distribution often have coverage substantially below .95.

## 7 EXAMPLE

**Figure 7:**
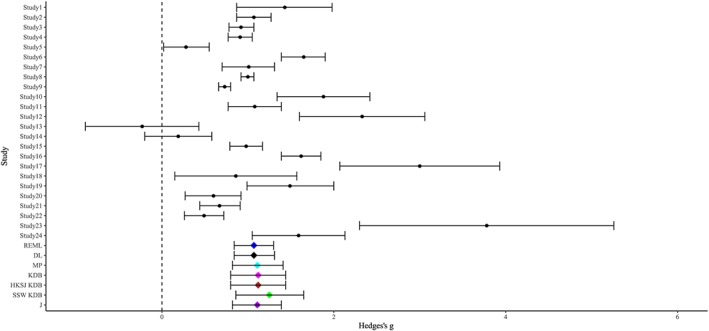
Forest plot of Hedges's *g* for the efficacy of psychological treatments for obsessive‐compulsive disorder [Colour figure can be viewed at http://wileyonlinelibrary.com]

As an example, we use data previously considered by Sánchez‐Meca and Marín‐Martínez,^35^ on the efficacy of psychological treatments for obsessive‐compulsive disorder (OCD). These data, Table 4, consist of 24 trials with mostly small sample sizes, ranging from 12 to 121 patients. Studies 5, 15, 16, and 23 are rather unbalanced; study 5 has 23 patients in the treatment arm and 11 in the control arm. The effect measure is SMD, and positive values correspond to lower levels of obsessions and compulsions in the treatment group. Figure 7 shows a forest plot, and Table 5 gives the results from various methods of estimation; recommended choices are in bold.

**Table 4:** Data for the meta‐analysis on the efficacy of psychological treatments for obsessive‐compulsive disorder. Design of study: 1, quasi‐experimental; 2, experimental

Study	Year	Design	*n*_*iT*_	*n*_*iC*_	*g*_*i*_	$v_{i}^{2}$
1	1998	1	10	8	1.425	0.2814
2	2003	2	22	23	1.068	0.1016
3	1993	2	29	32	0.924	0.0727
4	1993	2	29	32	0.909	0.0725
5	2005	1	23	11	0.281	0.1355
6	2005	2	21	20	1.646	0.1307
7	1997	2	15	14	1.007	0.1556
8	2002	2	55	66	0.996	0.0374
9	2002	2	55	66	0.731	0.0355
10	1998	2	11	10	1.882	0.2752
11	2000	2	13	16	1.082	0.1596
12	1997	2	9	9	2.326	0.3725
13	1994	2	6	6	−0.229	0.3355
14	1980	2	10	10	0.191	0.2009
15	2001	2	18	33	0.980	0.0953
16	2001	2	16	33	1.620	0.1196
17	2005	2	10	8	2.997	0.4745
18	1999	1	6	6	0.860	0.3642
19	2006	2	10	10	1.494	0.2558
20	2003	1	11	15	0.597	0.1644
21	1998	2	19	16	0.674	0.1216
22	1998	2	19	16	0.490	0.1186
23	2004	2	6	9	3.780	0.7541
24	2004	2	10	9	1.590	0.2776

**Table 5:** Point and confidence‐interval estimates for *τ* ^2^ and *δ* in the example of efficacy of psychological treatments for obsessive‐compulsive disorder; FE is fixed‐effect model and RE is random‐effects model. The heterogeneity parameter in RE is *τ* ^2^. *L* and *U* denote the lower and upper limits of the 95% confidence intervals. Recommended estimators in bold

Model	Method	${\hat{\tau }}^{2}$	*L*	*U*	$\hat{\delta }$	*L*	*U*	Length
								of CI
FE					0.9926	0.8516	1.1336	0.2820
RE	DL&IV	0.1697	0.0991	1.1002	1.0748	0.8431	1.3065	0.4634
RE	BJ&IV		0.0494	0.5128				
RE	J&IV	0.3275	0.1315	0.8214	1.1059	0.8215	1.3903	0.5688
RE	REML&IV	0.1622	0	0.6028	1.0728	0.8440	1.3016	0.4576
RE	MP&IV	**0.3722**	**0.0991**	**1.1002**	1.1122	0.8149	1.4095	0.5946
RE	KDB&IV	**0.4539**	0.2162	0.9052	1.1221	0.8027	1.4414	0.6387
RE	HKSJ (DL)				1.0748	0.7850	1.3646	0.5796
RE	HKSJ KDB				1.1221	0.8023	1.4418	0.6395
RE	SSW&KDB				**1.0950**	**0.7002**	**1.4898**	0.7896

Abbreviations: BJ, Biggerstaff and Jackson; DL, DerSimonian‐Laird; HKSJ, Hartung‐Knapp‐Sidik‐Jonkman; J, Jackson; KDB, Kulinskaya‐Dollinger‐Bjørkestøl; MP, Mandel‐Paule; REML, restricted maximum‐likelihood.

The estimated values of *τ* ^2^ have almost a threefold range, from 0.16 for REML to 0.45 for KDB. The methods differ much less in estimation of SMD. To a large degree, this is due to the relatively large number of studies (24). For instance, the variance of the overall effect for SSW given by (12) includes the multiplier of $\sum {\tilde{n}}_{i}^{2}/{\left(\sum {\tilde{n}}_{i}\right)}^{2}$ for *τ* ^2^, and it is clearly of the order 1/*K* (equal to 1/*K* for equal sample sizes $\tilde{n}$). This is also true for other estimators, so the differences between point estimators of *δ* almost disappear.

The results of our simulations for small sample sizes and *δ* near 1, Figures H1 and H2 in Supplementary Materials, indicate that *τ* ^2^ may be somewhat overestimated by KDB, somewhat underestimated by MP, and even more underestimated by REML, J, and especially DL. Combining this information with the results in Table 5, we expect *τ* ^2^ ≥ 0.4, much higher than the value of ${\hat{\tau }}_{DL}^{2}(=0.1697)$. On the other hand, the Q‐profile method is expected to provide the best confidence interval for *τ* ^2^, here (0.099,1.100), whereas the KDB interval may be too narrow at (0.216,0.905). Both confidence intervals include a sizable range of values of *τ* ^2^.

For *δ*, we expect all standard methods to yield negatively biased point estimates, including the KDB‐based IV‐weighted estimate at 1.122, so the SSW estimate of 1.095 seems somewhat low. From our simulations, the two best confidence intervals for *δ* are HKSJ KDB, here (0.802,1.442), and the DL‐based HKSJ, here (0.785,1.365), but both may be too narrow. The SSW KDB interval, centered at the SSW point estimator, with ${\hat{\tau }}_{\text{KDB}}^{2}$ in its estimated variance and t critical values, is widest, at (0.700,1.490); it may be too conservative, because it is 1.235 times as wide as HKSJ KDB and 1.362 times as wide as HKSJ.

**Figure 8:**
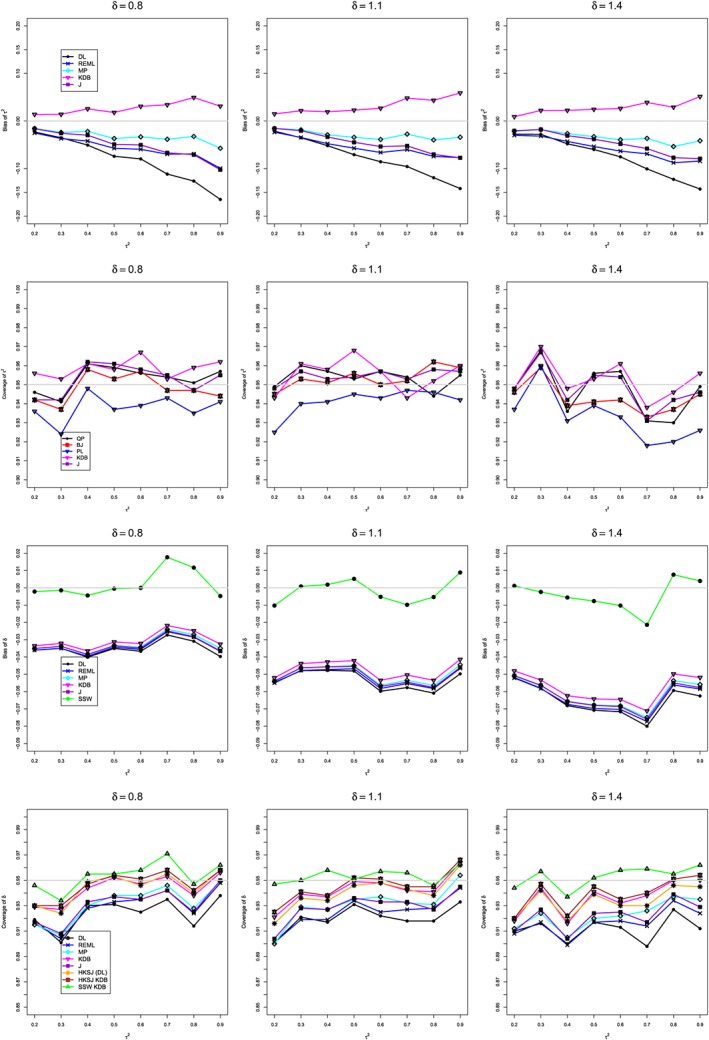
Quality of meta‐analysis methods for bias of *τ* ^2^, coverage of *τ* ^2^, bias of *δ* and coverage of *δ* with typical values of *τ* ^2^ and *δ* from the obsessive‐compulsive disorder example (*δ*=0.8,1.1,1.4 and *τ* ^2^∈[0.2,0.9]) and sample sizes *n*_*iT*_ and *n*_*iC*_ shown in Table 4. DL, DerSimonian‐Laird; HKSJ, Hartung‐Knapp‐Sidik‐Jonkman; J, Jackson; KDB, Kulinskaya‐Dollinger‐Bjørkestøl; MP, Mandel‐Paule; REML, restricted maximum‐likelihood [Colour figure can be viewed at http://wileyonlinelibrary.com]

We performed a small simulation (1000 replications per configuration), using values of *τ* ^2^ and *δ* within the confidence limits in Table 5. The results, plotted in Figure 8, show that the KDB method yields the least‐biased estimates of *τ* ^2^ and has coverage of *τ* ^2^ comparable to or better than other methods. However, for these data, we prefer the more conservative QP interval. The HKSJ KDB interval for *δ* provides the best, though still somewhat liberal, coverage of *δ* among all intervals centered at an IV‐weighted estimate. As expected, the sample‐size‐weighted estimator of *δ* is the only unbiased estimator, and the SSW KDB interval provides the most reliable though sometimes conservative coverage of *δ*. These methods are our recommended choices for estimation of *δ*.

## 8 DISCUSSION: PRACTICAL IMPLICATIONS FOR META‐ANALYSIS

Methods for random‐effects meta‐analysis require an estimate of the between‐study variance, *τ* ^2^. We show that the performance of the popular estimators of *τ* ^2^ and related estimators of the overall effect varies widely among effect measures, and the existing evidence is scarce. For the effect measures MD and SMD, we use improved effect‐measure‐specific approximations to the expected value and distribution of *Q* to introduce two new methods of point estimation of *τ* ^2^ for MD (WT and CDL) and one WT interval method. We introduce one point estimator and one interval estimator for *τ* ^2^ in SMD. We also provide the first comprehensive simulation study for both MD and SMD.

The results of our simulations give a rather disappointing picture of the current state of meta‐analysis for most common measures of effect. In brief, small sample sizes are rather problematic for many methods of meta‐analysis, even for such a well‐behaved effect measure as the MD, and meta‐analyses that involve numerous small studies are especially challenging.

For MD, the between‐study variance, *τ* ^2^, is usually overestimated near zero. When *n*=20, DL has a constant positive bias of about 0.07 regardless of *τ* ^2^. REML is better for larger *τ* ^2^, but it is about the same for *τ* ^2^ ≤ 0.2 when *K*=30. These are the main methods used in the vast majority of meta‐analyses. MP is the best at 0.03 to 0.06 bias (Figure 1). We do not recommend the WT point estimator of *τ* ^2^. The CDL point estimator of *τ* ^2^ is essentially unbiased when *n*=40, and it is the most reliable overall, across all values of *τ* ^2^, *n*, and *K*; and our WT method provides reliable interval estimation. The estimators of *μ* are unbiased. Widespread complacency about the quality of meta‐analysis methods is due to the use of MD as the outcome measure in many simulations. HKSJ intervals provide good but too liberal coverage of MD when studies are small and/or unbalanced. Our SSW CDL intervals are more reliable in this case, especially for larger *K*.

Arendacká^36^ and Liu et al.^37^ propose new confidence intervals for *τ* ^2^ in the one‐way heteroscedastic random‐effects model. These intervals can be used directly in meta‐analysis of means in noncomparative studies. Both publications include extensive simulations and compare their intervals with those of Knapp et al.^15^ Both proposals seem to do very well for normal distributions and very small sample sizes. It should be possible to extend these methods to MD in comparative two‐arm designs; we plan to pursue this extension elsewhere.

For other effect measures, the picture is much more concerning. Because the study‐level effects and their variances are related (as in Equation  (3) for SMD), the performance of all statistical methods depends on the effect measures, estimates of overall effects are biased, and coverage of confidence intervals is too low, especially for small sample sizes. We see this for SMD. Bias of all inverse‐variance methods for SMD when *n*=20 is about 7% (Figure 4). Coverage of SMD is considerably worse when SMD is large and *τ* ^2^<0.5, at about 85% for HKSJ (Figure 6). This may easily lead to misinterpretation of clinical findings.

The conventional wisdom is that these deficiencies do not matter, as meta‐analysis usually deals with studies that are “large,” so all these little problems are automatically resolved. Unfortunately, this is not true, even in medical meta‐analyses; in Issue 4 of the Cochrane Database 2004, the maximum study size was 50 or less in 25% of meta‐analyses that used MD as an effect measure, and less than 110 in 50% of them.^38^ We have not surveyed typical study sizes in psychology, but Sánchez‐Meca and Marín‐Martínez,^35^ promoting MA in psychological research, use an example with 24 studies in which the smallest study size is 12 and the largest is 121. We considered this example in Section 7. In ecology, typical sample sizes are between 4 and 25.^39^ An effect‐measure‐specific estimator of *τ* ^2^, such as KDB for SMD, can reduce inherent biases.

Arguably, the main purpose of a meta‐analysis is to provide point and interval estimates of an overall effect. Usually, after estimating the between‐study variance *τ* ^2^, inverse‐variance weights are used in estimating the overall effect (and, often, its variance). This approach relies on the theoretical result that, for known variances, and given unbiased estimates ${\hat{\theta }}_{i}$, it yields a uniformly minimum‐variance unbiased estimate (UMVUE) of *θ*. In practice, however, the true within‐study variances are unknown, and use of the estimated variances makes the inverse‐variance‐weighted estimator of the overall effect biased. Consumers routinely expect point estimates to have no (or small) bias and CIs to have (close to) nominal coverage. Thus, the IV‐weighted approach is unsatisfactory because, in general, it cannot produce an unbiased estimate of an overall effect.

We agree with Rukhin^40^: “A meta‐analyst must be willing to use different estimates of the between‐study variance *σ* ^2^ for different purposes: one to minimize the variance of the treatment effect statistic; another to construct a reliable confidence interval for this parameter; yet another to estimate *σ* ^2^ itself!” Our recommendations for meta‐analysis of MD and SMD appear in Table 3.

A pragmatic approach to unbiased estimation of *δ* uses weights that do not involve estimated variances of study‐level estimates, for example, weights proportional to the study sizes *n*_*i*_. Hunter and Schmidt^29^ and Shuster,^41^ among others, have proposed such weights, and Marín‐Martínez and Sánchez‐Meca^42^ and Hamman et al^39^ have studied the method's performance by simulation for SMD. We prefer to use weights proportional to an effective sample size, ${\tilde{n}}_{i}=n_{iT}n_{iC}/n_{i}$; these are the optimal inverse‐variance weights for SMD when *δ*=0 and *τ* ^2^=0. Thus, the overall effect is estimated by ${\hat{\theta }}_{SSW}=\sum {\tilde{n}}_{i}{\hat{\theta }}_{i}/\sum {\tilde{n}}_{i}$, and its variance is estimated by Equation (12). Hamman et al^39^ use weights proposed by Hedges,^43^ which differ slightly from $\tilde{n}$ for very small sample sizes. A good estimator of *τ* ^2^, such as MP or KDB (for SMD), can be used as ${\hat{\tau }}^{2}$. Furthermore, confidence intervals for *θ* centered at ${\hat{\theta }}_{\text{SSW}}$ with ${\hat{\tau }}_{KDB}^{2}$ in Equation (12) can be used.

This approach based on SSW requires further study. For example, in the confidence intervals, we have used critical values from the *t*‐distribution on *K*−1 degrees of freedom, but we have not yet examined the actual sampling distribution of SSW. The raw material for such an examination is readily available: For each situation in our simulations, each of the 10 000 replications yields an observation on the sampling distribution of SSW.

## Supporting information

SIM_8422‐Supp‐0001.zip
