# Supplementary material for: Estimation in meta‐analyses of mean difference and standardized mean difference
Source: Stat Med. 2019 Nov 11;39(2):171–91. doi: 10.1002/sim.8422 (PMC6916299; doi:10.1002/sim.8422)
Supplement: Supplementary file 1 — SIM_8422‐Supp‐0001.zip [file SIM-39-171-s001.zip › MD_SMD_WebAppendix_A0_13Aug19.pdf]

APPENDIX

Web Appendix A

for

Ilyas Bakbergenuly, David C. Hoaglin, and Elena Kulinskaya

Estimation in meta-analyses of mean difference and standardized mean difference

Previous simulation studies of MD and SMD

Table A1 lists previous simulation studies for MD. Only two studies (Viechtbauer[1] and Petropoulou at al.[2]) consider and compare several point estimators of τ^2. Knapp et al.[3] consider quality of interval estimation. Unfortunately, these and other studies (wrongly) use the pooled estimator of the variance of MD. The pooled estimator is equivalent to the unpooled estimator, given by Equation (2.1), only when the sample sizes are equal within the study. Simulations that generate one normally distributed study-level effect and χ^2-distributed sample variance[4] are equivalent to simulations with the pooled variance.

Table A2 lists previous simulation studies for SMD. Only three studies (Viechtbauer[1], Petropoulou et al.[2], and Langan et al.[5]) consider and compare several point estimators of τ^2. Viechtbauer[1] provides conclusions on point estimation of τ^2 for MD and SMD. For SMD Langan et al.[5] study bias and mean squared error of estimators of τ^2, as well as coverage of confidence intervals for the overall effect, but they use only one value of the true SMD ($\delta = 0.5$) because they believe that the value of δ does not matter. Our simulations show that this is incorrect.

Novianti et al.[6] study SMD in sequential meta-analysis, which may differ greatly from the setting of the standard random-effects model. Consistent with our view, they comment on the very different performance of the DerSimonian-Laird method[7] (DL) for log-odds-ratio and SMD. Chung et al.[8] and Jackson[9] consider point or interval estimation of τ^2 for MD, but they generate within-study sample variances from a truncated and scaled χ^2_1 distribution, following an approach that Brockwell and Gordon[10] used for log-odds-ratio. That approach, however, ignores the fact that for MD the sample variance is a weighted mean of chi-squared random variables whose degrees of freedom are defined by the studies' sample sizes.

In meta-analyses of MD and SMD, Petropoulou and Mavridis[2] use simulation to study 20 estimators of heterogeneity variance and their impact on coverage and length of 95% confidence intervals for the overall effect, but they use the pooled variance for MD. To assess bias of the estimators of heterogeneity variance, they use mean absolute error (the only performance measure reported for SMD in their Table S8), which is not a measure of bias; it is the linear counterpart of mean squared error.

Several studies have considered the quality of estimation of various effect measures, but not estimation of heterogeneity variance. Friedrich et al.[11] report extensive simulations for MD and SMD, but they use only the DL method to estimate τ^2 and do not report on its quality. Lin[12] provides similar simulations for MD and SMD. IntHout et al.[13] study coverage for MD and combinations of different-sized studies, using the standard method and the Hartung-Knapp-Sidik-Jonkman (HKSJ) method based on the DL estimator of τ^2 (Hartung and Knapp[14], Sidik and Jonkman[15]). For MD, Partlett and Riley[4] provide another in-depth study of coverage based on restricted-maximum-likelihood (REML) estimation of τ^2. They also recommend HKSJ confidence intervals. Hamman et al.[16] study the quality of estimation of SMD using REML to estimate τ^2 and inverse-variance weights, inverse-sample-size weights, and no weights. They recommend using fixed weights, noting considerable biases of the inverse-variance-weighted method. Veroniki et al.[17] report on a comprehensive survey of interval estimators for the overall effect in random-effects meta-analysis; they also survey comparative studies that use simulation and real-life data. They found only four simulation studies for MD, and six for SMD, mostly cited above.

The range of values of μ_i in previous simulation studies of MD varies from 0 in Viechtbauer[1] to 56 in Friedrich et al.[11], but the value of μ_i is unimportant because $\hat{\sigma}_i^2$ does not involve μ_i. In Viechtbauer[1] τ^2 and the σ_i^2 are commeasurable and vary from 0.0625 to 1. IntHout et al.[13] set $\tau^2 = C\bar{\sigma}^2$, where $\bar{\sigma}^2$ is the average within-study variance, and vary C from 1/3 to 9. In Friedrich et al.[11] the τ^2 values, if non-zero, are much larger at $\tau^2 = n\sigma^2/4$ for $n = 10$ and 100. Partlett and Riley[4] vary τ^2 from 0.01 to 1 and express it as a multiple of $\sigma_i^2 = 0.1$.

For simulations of SMD Viechtbauer[1] and Friedrich et al.[11] use $\delta = 0.2$, 0.5, and 0.8, designated by Cohen[18] as small, medium, and large effect sizes. These categories, however, are not universal. Hamman et al.[16] use δ from 0 to 2.5 and τ^2 from 0 to 10 as relevant ranges for ecology. Therefore, our simulation study for SMD uses $0 \leq \delta \leq 2$ and $0 \leq \tau^2 \leq 2.5$ as realistic for a range of applications.

Study	(σ_T^2, σ_C^2)	μ	τ^2	n and/or $\bar{n}$ *	K	$\hat{\tau}^2$	τ^2 intervals	$\hat{\mu}$	μ intervals
Viechtbauer 2005	(10, 10)<br>pooled variance	0,1,2,4	0,0.125,0.25,0.5,1	$\bar{n} = 20, 40, 80, 160, 320$<br>$n_i \sim N(\bar{n}, (\bar{n}/3)^2)$<br>$n_{iT} = n_{iC} = n_i$	5, 10,<br>20, 40, 80	DL<br>ML<br>REML<br>HE<br>HS			
Knapp et al. 2006	100, 10 across studies<br>pooled variance	0	1, 2.5, 5, 10, 20	20, 40	5, 10, 20, 50, 100		KBH		
Friedrich et al 2008	$\sigma_T = \sigma_C = \sigma = 10,\ 40,\ 70$	$0.2\sigma,\ 0.5\sigma,\ 0.8\sigma$	$0,\ 0.5\sigma$	$n = 10,\ 100, n_T = n_C$;<br>$n_T = n_C/2,\ n_T = 2n_C$<br>(with $n_T + n_C = 2n$)	5, 10, 30	DL		IV	IV
Lin 2018	$\sigma_{iT} = \sigma_{iC} \sim U(1,5)$<br>pooled variance	0, 0.5, 1, 2, 5	0, 0.25, 1	U(5,10), U(10,20),<br>U(20,30),U(30,50)<br>U(50,100),U(100,500),<br>U(500,1000); $n_{Ti} = n_{Ci} = n_i/2$	5, 10, 20, 50	DL		IV	IV
IntHout et al. 2014	(1,1)<br>pooled variance	0	depends on I^2 and the n_i,<br>I^2 =0, 25%, 50%, 75%, 90%	$n_T = n_C = n = 25,\ 50,$<br>100, 250, 500, 1000<br>$\bar{n}$=100, 250, 500, 1000;<br>25%, 50%, 75% large (=10× small)	2(1)10(5)20	DL		IV	IV<br>HKSJ
Partlett and Riley 2017	one normal mean<br>$\sigma^2 = 0.1$	1	0.01, 0.05, 0.1, 1	30	3, 5, 7, 10, 100	REML		IV	IV<br>KR<br>HKSJ, HK2<br>SJ, SJ2
Petropoulou and Mavridis 2017	(1,1)<br>pooled variance	0, 0.5	0, 0.01, 0.05, 0.5<br>$\mu_i \sim N(\mu, \tau^2)$<br>$\mu_i \sim$ Laplace(0, 1)	$n_T = n_C \sim U(20,\ 200)$	5, 10, 20, 30	17<br>estimators<br>of τ^2		IV	IV
Jackson 2013	one normal mean & known variances<br>$(\sigma_1^2, \ldots, \sigma_5^2) =$<br>(0.009, 0.046, 0.122, 0.265, 0.600)	0	0, 0.029, 0.069, 0.206, 1.302	implicit in σ_i^2	5; 10, 20, 40	DL	BJ<br>J<br>QP		

TABLE A1 Simulation studies on meta-analysis of MD.
Point estimators of τ^2: DL - DerSimonian-Laird, ML - Maximum likelihood, REML - Restricted maximum likelihood, HE - Hedges, HS - Hunter and Schmidt.
Interval estimators fof τ^2: QP - Q-profile, BJ - Biggerstaff and Jackson, J - Jackson, KBH - Knapp et al. (2006).
Point estimators of μ: IV - inverse-variance-weighted;
Interval estimators of μ: all confidence intervals are centered at an IV estimator of μ. IV with z quantiles: HKSJ, HK2, SJ, SJ2 and KR. Confidence intervals using t quantiles with variance estimators by: HKSJ: Hartung and Knapp (2001), Sidik and Jonkman(2002); HK2: Röver et al. (2015); SJ and SJ2: Sidik and Jonkman (2006); KR: Kenward and Roger (1997).
* $\bar{n}$ is the average sample size used when sample sizes differ across studies.

Study	SMD measure	δ	τ^2	n and/or $\bar{n}$ *	K	$\hat{\tau}^2$	Coverage of $\hat{\tau}^2$	$\hat{\delta}$	Coverage of $\hat{\delta}$
Viechtbauer et al. 2005	Hedges′s d	0,0.2,0.5,0.8	0,0.01,0.025,0.05,0.1	$\bar{n} = 20, 40, 80, 160, 320$	5,10<br>,20,40,80	DL<br>ML<br>REML<br>HE<br>HS		IV	
Friedrich et al 2008	Hedges′s d	0.2,0.5,0.8	0,0.5	$n_T = n_C = 10, 100$	5,10,30	DL		IV	IV
Petropoulou and Mavridis (2017)		0 & 0.5	0,0.01,0.05,0.5	$n_T = n_C\ U(20,\ 200)$	5,10,20, 30	20 different estimators of τ^2		IV	IV
Langan et al. 2018	Hedges′s d	0.5	depends of I^2, 0%,15%<br>30%,45%,60%,<br>75%,90%,95%	N=40, N=U(40,400),<br>N=400, N=U(2000,4000),<br>N=400+N=U(2000,4000)	2,3,5,<br>10,20,30,<br>50,100	REML<br>CA<br>PM or MP<br>PM_{CA}<br>PM_{DL}<br>HM<br>SJ<br>SJ_{CA}		IV	IV<br>IV+t<br>IV+t +HKSJ
Lin 2018	Cohen′s d<br>Hedges′s d	0,0.2,0.5,0.8,1	0,0.2,0.5	U(5,10), U(10,20),<br>U(20,30),U(30,50)<br>U(50,100),U(100,500),<br>U(500,1000)	5,10,20,50	DL		IV	IV
Hamman et al. 2018	Hedges′s d	0,0.1,0.15,0.25<br>0.35,0.5,0.6,0.75,<br>1,1.25,1.5,2.5	0,0.1,0.5,1,<br>2.5,5,10	$\bar{n} = 4, 6, 8, 10, 12,$<br>14,16,20,25	5,10,15,25,35,<br>45,55,75,100,125	REML		IV<br>SSW(H)<br>EW	IV+t +HKSJ
Marín-Martínez and<br>Sánchez-Meca 2010<br>Sánchez-Meca 2010		0.2,0.5,0.8	0,0.04,0.08,0.16,0.32	$\bar{n} = 30, 50, 80, 100$	5,10,20,40,100	DL<br>ML<br>ML		FE<br>IV<br>HS	

TABLE A2 Simulation studies on meta-analysis of SMD.

Estimators of $\hat{\tau}^2$: DL - DerSimonian and Laird estimator, ML - Maximum likelihood, REML - Restricted maximum likelihood estimator, HE - Hedges estimator, HS - Hunter-Schmidt estimator, CA - Cochran Anova, PM or MP - Mandel-Paule estimator, PM_{CA} - two-step Cochran Anova, PM_{DL} -two-step DerSimonian-Laird, HM - Hartung-Makambi, SJ - Sidik Jonkman, SJ_{CA} - alternative Sidik-Jonkman, BM - Bayes Modal estimator;

Estimators of δ: SSW(H) - sample size weighted (Hedges, 1983a), EW - equal weights, IV - inverse-variance estimator, HS - Hunter and Schmidt (1990,2004) total sample size weighted;

Coverage of $\hat{\delta}$: IV - confidence interval centered at inverse variance estimator of $\hat{\delta}$ with z-quantiles , IV+t - confidence interval centered at inverse variance estimator of $\hat{\delta}$ with t quantiles, IV+HKSJ - Hartung-Knapp-Sidik-Jonkman estimator centered at inverse variance estimator of $\hat{\delta}$.

* $\bar{n}$ is the average sample size used when sample sizes differ across studies.

References

1. Viechtbauer W. Bias and efficiency of meta-analytic variance estimators in the random-effects model. *Journal of Educational and Behavioral Statistics* 2005; 30(3): 261–293.

2. Petropoulou M, Mavridis D. A comparison of 20 heterogeneity variance estimators in statistical synthesis of results from studies: a simulation study. *Statistics in Medicine* 2017; 36(27): 4266–4280.

3. Knapp G, Biggerstaff BJ, Hartung J. Assessing the amount of heterogeneity in random-effects meta-analysis. *Biometrical Journal* 2006; 48(2): 271–285.

4. Partlett C, Riley RD. Random effects meta-analysis: coverage performance of 95% confidence and prediction intervals following REML estimation. *Statistics in Medicine* 2017; 36(2): 301–317.

5. Langan D, Higgins JPT, Jackson D, et al. A comparison of heterogeneity variance estimators in simulated random-effects meta-analyses. *Research Synthesis Methods* 2019; 10(1): 83–98.

6. Novianti PW, Roes KCB, van der Tweel I. Estimation of between-trial variance in sequential meta-analyses: a simulation study. *Contemporary Clinical Trials* 2014; 37(1): 129–138.

7. DerSimonian R, Laird N. Meta-analysis in clinical trials. *Controlled Cinical Trials* 1986; 7(3): 177–188.

8. Chung Y, Rabe-Hesketh S, Choi IH. Avoiding zero between-study variance estimates in random-effects meta-analysis. *Statistics in Medicine* 2013; 32(23): 4071–4089.

9. Jackson D. Confidence intervals for the between-study variance in random effects meta-analysis using generalised Cochran heterogeneity statistics. *Research Synthesis Methods* 2013; 4(3): 220–229.

10. Brockwell SE, Gordon IR. A comparison of statistical methods for meta-analysis. *Statistics in Medicine* 2001; 20(6): 825–840.

11. Friedrich JO, Adhikari NK, Beyene J. The ratio of means method as an alternative to mean differences for analyzing continuous outcome variables in meta-analysis: a simulation study. *BMC Medical Research Methodology* 2008; 8: 32.

12. Lin L. Bias caused by sampling error in meta-analysis with small sample sizes. *PLoS ONE* 2018; 13(9): e0204056.

13. IntHout J, Ioannidis JPA, Borm GF. The Hartung-Knapp-Sidik-Jonkman method for random effects meta-analysis is straightforward and considerably outperforms the standard DerSimonian-Laird method. *BMC Medical Research Methodology* 2014; 14: 25.

14. Hartung J, Knapp G. A refined method for the meta-analysis of controlled clinical trials with binary outcome. *Statistics in Medicine* 2001; 20(24): 3875–3889.

15. Sidik K, Jonkman JN. A simple confidence interval for meta-analysis. *Statistics in Medicine* 2002; 21(21): 3153–3159.

16. Hamman EA, Pappalardo P, Bence JR, Peacor SD, Osenberg CW. Bias in meta-analyses using Hedges' d. *Ecosphere* 2018; 9(9): e02419. doi: 10.1002/ecs2.2419

17. Veroniki AA, Jackson D, Bender R, et al. Methods to calculate uncertainty in the estimated overall effect size from a random-effects meta-analysis. *Research Synthesis Methods* 2018; to appear.

18. Cohen J. *Statistical Power Analysis for the Behavioral Sciences*. New York: Academic Press . 1988.
