# Supplementary material for: Estimation in meta‐analyses of mean difference and standardized mean difference
Source: Stat Med. 2019 Nov 11;39(2):171–91. doi: 10.1002/sim.8422 (PMC6916299; doi:10.1002/sim.8422)
Supplement: Supplementary file 1 — SIM_8422‐Supp‐0001.zip [file SIM-39-171-s001.zip › MD_SMD_WebAppendix_B0.pdf]

APPENDIX

Web Appendix B

for

Ilyas Bakbergenuly, David C. Hoaglin, and Elena Kulinskaya

Estimation in meta-analyses of mean difference and standardized mean difference

COMPARATOR METHODS OF ESTIMATING BETWEEN-STUDY VARIANCE

B1 POINT ESTIMATORS

B1.1 DerSimonian-Laird method (DL)

When $\tau^2 = 0$, Cochran's Q-statistic $Q = \sum \hat{w}_i(\hat{\theta}_i - \hat{\theta})^2$, with $\hat{w}_i = \hat{w}_i(0) = 1/\hat{\sigma}_i^2$ and $\hat{\theta} = \sum_{i=1}^{K} \hat{w}_i\hat{\theta}_i / \sum_{i=1}^{K} \hat{w}_i$, is customarily assumed to have approximately the chi-squared distribution χ^2_{K-1}. DerSimonian and Laird[1] substitute $w_i = 1/\sigma_i^2$ for $\hat{w}_i$, derive the corresponding expected value of Q when $\text{Var}(\hat{\theta}_i) = \sigma_i^2 + \tau^2$, and estimate τ^2 by the method of moments:

$$\hat{\tau}^2_{DL} = \max\left(0, [Q - K + 1] \Big/ \left[\sum_{i=1}^{K} \hat{w}_i - \frac{\sum_{i=1}^{K} \hat{w}_i^2}{\sum_{i=1}^{K} \hat{w}_i}\right]\right). \quad \text{(B1)}$$

B1.2 Restricted-maximum-likelihood method (REML)

Assuming that the $\hat{\theta}_i$ are distributed as $N(\theta, \hat{\sigma}_i^2 + \tau^2)$, the restricted-maximum-likelihood (REML) estimator $\hat{\tau}^2_{REML}$ maximizes the restricted (or residual) log-likelihood function $l_R(\theta, \tau^2)$. It is obtained iteratively from

$$\hat{\tau}^2_{REML} = \frac{\sum_{i=1}^{K} [\hat{w}_i(\hat{\tau}^2)]^2 [(\hat{\theta}_i - \hat{\theta}_{REML})^2 - \hat{\sigma}_i^2]}{\sum_{i=1}^{K} [\hat{w}_i(\hat{\tau}^2)]^2} + \frac{1}{\sum_{i=1}^{K} \hat{w}_i(\hat{\tau}^2)}, \quad \text{(B2)}$$

and truncated at zero, for $\hat{\theta}_{REML}$ given by Equation (3.2) with weights $\hat{w}_i(\hat{\tau}^2_{REML})$. REML is preferred to the maximum-likelihood estimator of τ^2 because the latter fails to account for the simultaneous estimation of θ and it is superior to DL because of its balance between unbiasedness and efficiency[2]. However, like DL, using the $\hat{\sigma}_i^2$ as if they were the σ_i^2 may undermine its performance.

One can also obtain the REML estimator of τ^2 by maximizing the penalized log-likelihood developed by Kosmidis et al.[3] to reduce the bias of maximum-likelihood estimation.

B1.3 Jackson method (J)

DerSimonian and Kacker[4] generalized Q, replacing the $\hat{w}_i$ by arbitrary fixed positive constants, a_i, and obtaining $Q_a = \sum a_i(\hat{\theta}_i - \hat{\theta}_a)^2$, from which they derived a general method-of-moments estimator of τ^2. They discussed several special cases, including DL (with $a_i = 1/\hat{\sigma}_i^2$, treating the $\hat{\sigma}_i^2$ as fixed).

As an option when there is little a priori knowledge about the extent of heterogeneity, but some is anticipated, Jackson[5] proposed the interval estimator of τ^2 based on Q_a with $a_i = 1/\sigma_i$. His computational procedure avoids negative $\hat{\tau}^2$. The R

function *inference* in the supplementary materials to Jackson[5] returns the corresponding point estimate. We abbreviate the point and interval estimators as J. Actual meta-analyses would use the $\hat{\sigma}_i$, so the a_i in Q_a are not fixed.

B1.4 Mandel-Paule method (MP)

The Mandel-Paule (MP) estimator, $\hat{\tau}^2_{MP}$, is another moment-based estimator of the between-study variance. For measurements made in K laboratories (n_i measurements per laboratory, with common within-laboratory variance σ^2_W), Mandel and Paule[6] developed an iterative method of estimating the between-laboratory variance component, σ^2_B, for the random laboratory effects. Defining $\lambda = \sigma^2_B/\sigma^2_W$, $\omega_i = 1/(\lambda + 1/n_i)$, and $\hat{\mu} = \Sigma\omega_i\bar{y}_i/\Sigma\omega_i$, they observe that (when σ^2_B and σ^2_W are known) the expected value of $\Sigma\omega_i(\bar{y}_i - \hat{\mu})^2$ is $(K-1)\sigma^2_W$, corresponding to the expected value when $\sigma^2_B = 0$. A straightforward iteration, with $\hat{\sigma}^2_W$ in place of σ^2_W, yields the desired value of $\hat{\lambda}$ and hence $\hat{\sigma}^2_B = \hat{\lambda}\hat{\sigma}^2_W$. Paule and Mandel[7] allow separate within-laboratory variances, σ^2_{Wi} (estimated by s^2_{Wi}), and use weights $\hat{\omega}_i = \left[(s^2_{Wi}/n_i) + s^2_B\right]^{-1}$. They define

$$F(s^2_B) = \Sigma\hat{\omega}_i(\bar{y}_i - \hat{\mu})^2 - (K-1)$$

(in which $\hat{\mu}$ is based on the $\hat{\omega}_i$) and use the truncated Taylor-series expansion

$$F(s^2_B) \approx F_0 + \left(\frac{\partial F}{\partial s^2_B}\right)_0 ds^2_B,$$

with

$$\left(\frac{\partial F}{\partial s^2_B}\right) = -\Sigma\hat{\omega}^2_i(\bar{y}_i - \hat{\mu})^2,$$

to iterate to the solution $F(\hat{s}^2_B) = 0$. (The partial derivative is < 0, so F is monotonically decreasing.)

Apart from the $K-1$, the F of Paule and Mandel differs from the Q of meta-analysis by using the random-effects weights instead of the fixed-effect weights. Thus, as in Section 3, the random-effects weights and $\hat{\theta}_{RE}$ depend on τ^2; we denote the resulting Q by $Q(\tau^2)$ and obtain $\hat{\tau}^2_{MP}$ by iteratively solving the equation

$$Q(\tau^2) = \sum_{i=1}^{K} \hat{w}_i(\tau^2)(\hat{\theta}_i - \hat{\theta}_{RE})^2 = K - 1 \quad \text{(B3)}$$

and requiring $\hat{\tau}^2_{MP} > 0$.

This method is equivalent to the empirical Bayes methods of Carter and Rolph[8] and Morris[9], as noted by Rukhin and Vangel[10] and Rukhin et al.[11].

B2 INTERVAL ESTIMATORS

B2.1 Profile-likelihood interval (PL)

The 95% profile-likelihood confidence interval for τ^2 consists of the values that are not rejected by the likelihood-ratio test with τ^2 as the null hypothesis (Hardy and Thompson[14]). Here the other parameter in the likelihood, $\hat{\theta}$, is a function of τ^2. The values of τ^2 in the confidence interval satisfy

$$\left\{\tau^2 : \; l_R(\hat{\theta}(\tau^2), \tau^2) > l_R(\hat{\theta}_{REML}, \hat{\tau}^2_{REML}) - \frac{1}{2}\chi^2_{1;0.95}\right\}, \quad \text{(B4)}$$

where $\chi^2_{1;0.95} = 3.841$ is the 0.95 quantile of the χ^2_1 distribution, and $l_R(\hat{\theta}(\tau^2), \tau^2)$ is the restricted log-likelihood function evaluated at $(\hat{\theta}(\tau^2), \tau^2)$.

B2.2 Q-profile confidence interval (QP)

If the weight for Study i is $1/(\sigma^2_i + \tau^2)$, the generalized Q-statistic

$$Q(\tau^2) = \sum_{i=1}^{K} \frac{(\hat{\theta}_i - \hat{\theta}(\tau^2))^2}{\sigma^2_i + \tau^2} \quad \text{(B5)}$$

follows the chi-squared distribution with $K-1$ degrees of freedom. To obtain the Q-profile confidence interval, Viechtbauer[15] finds the lower and upper confidence limits by iteratively solving $Q(\tilde{\tau}_L^2) = \chi^2_{K-1;0.975}$ and $Q(\tilde{\tau}_U^2) = \chi^2_{K-1;0.025}$. In practice it is necessary to use the $\hat{\sigma}_i^2$ instead of the σ_i^2, and then the generalized Q-statistic no longer follows the assumed chi-squared distribution.

B2.3 Biggerstaff and Jackson interval (BJ)

For a generic effect measure, Biggerstaff and Jackson[16] derive the exact distribution of the statistic

$$Q = \sum_{i=1}^{K} w_i(\hat{\theta}_i - \hat{\theta})^2, \tag{B6}$$

where $w_i = 1/\sigma_i^2$ and $\hat{\theta} = (\sum w_i\hat{\theta}_i)/(\sum w_i)$. They show that the distribution is that of a linear combination of mutually independent chi-squared random variables, each with 1 degree of freedom, and they take advantage of available software for evaluating the cumulative distribution function F_Q of such a distribution.

That distribution yields a generalized Q-profile confidence interval, whose lower and upper limits are the solutions to the equations

$$Q(\tilde{\tau}_L^2) = F_{Q;0.975}, \qquad Q(\tilde{\tau}_U^2) = F_{Q;0.025}, \tag{B7}$$

in which $F_{Q;0.025}$ and $F_{Q;0.975}$ are, respectively, the 0.025 and 0.975 quantiles. If the equation for $\tilde{\tau}_L^2$ has no solution, they set $\tilde{\tau}_L^2 = 0$. We refer to this interval as the BJ confidence interval.

Despite the title of Biggerstaff and Jackson[16], Q in (B6) is not Cochran's heterogeneity statistic. In the definition of Q, Cochran[17] used $w_i = 1/\hat{\sigma}_i^2$.

B2.4 Jackson interval (J)

As mentioned in Section B1.3, Jackson[5] proposed another generalized Q-profile confidence interval for τ^2. The approach is the same as for the BJ interval, but with $a_i = 1/\sigma_i$ in Q_a.

References

1. DerSimonian R, Laird N. Meta-analysis in clinical trials. *Controlled Cinical Trials* 1986; 7(3): 177–188.
2. Viechtbauer W. Bias and efficiency of meta-analytic variance estimators in the random-effects model. *Journal of Educational and Behavioral Statistics* 2005; 30(3): 261–293.
3. Kosmidis I, Guolo A, Varin C. Improving the accuracy of likelihood-based inference in meta-analysis and meta-regression. *Biometrika* 2017; 104(2): 489–496.
4. DerSimonian R, Kacker R. Random-effects model for meta-analysis of clinical trials: an update. *Contemporary Clinical Trials* 2007; 28(2): 105–114.
5. Jackson D. Confidence intervals for the between-study variance in random effects meta-analysis using generalised Cochran heterogeneity statistics. *Research Synthesis Methods* 2013; 4(3): 220–229.
6. Mandel J, Paule RC. Interlaboratory evaluation of a material with unequal numbers of replicates. *Analytical Chemistry* 1970; 42(11): 1194–1197.
7. Paule RC, Mandel J. Consensus values and weighting factors. *Journal of Research of the National Bureau of Standards* 1982; 87(5): 377–385.
8. Carter G, Rolph J. Emprical Bayes Methods Applied to Estimating Fire Alarm Probabilities. *Journal of the American Statistical Association* 1974; 69: 880-885.
9. Morris C. Parametric empirical Bayes inference: Theory and applications. *Journal of the American Statistical Association* 1983; 78: 47-55.

10. Rukhin A, Vangel M. Estimation of a common mean and weighted means statistics. *Journal of the American Statistical Association* 1998; 93: 303–308.

11. Rukhin A, Biggerstaff B, Vangel M. Restricted Maximum Likelihood Estimation of a Common Mean and the Mandel-Paule Algorithm. *Journal of Statistical Planning and Inference* 2000; 83: 319–330.

12. Bowden J, Tierney J, Copas A, Burdett S. Quantifying, displaying and accounting for heterogeneity in the meta-analysis of RCTs using standard and generalised Q statistics. *BMC Medical Research Methodology* 2011; 11: 41.

13. Viechtbauer W, López-López J, Sánchez-Meca J, Marín-Martínez F. A Comparison of Procedures to Test for Moderators in Mixed-Effects Meta-Regression Models. *Psychological Methods* 2015; 20(3): 360-374.

14. Hardy RJ, Thompson SG. A likelihood approach to meta-analysis with random effects. *Statistics in Medicine* 1996; 15(6): 619–629.

15. Viechtbauer W. Confidence intervals for the amount of heterogeneity in meta-analysis. *Statistics in Medicine* 2007; 26(1): 37–52.

16. Biggerstaff BJ, Jackson D. The exact distribution of Cochran's heterogeneity statistic in one-way random effects meta-analysis. *Statistics in Medicine* 2008; 27(29): 6093–6110.

17. Cochran WG. The combination of estimates from different experiments. *Biometrics* 1954; 10(1): 101–129.
