# Supplementary material for: Estimation in meta‐analyses of mean difference and standardized mean difference
Source: Stat Med. 2019 Nov 11;39(2):171–91. doi: 10.1002/sim.8422 (PMC6916299; doi:10.1002/sim.8422)
Supplement: Supplementary file 1 — SIM_8422‐Supp‐0001.zip [file SIM-39-171-s001.zip › MD_SMD_WebAppendix_C0.pdf]

APPENDIX

Web Appendix C

for

Ilyas Bakbergenuly, David C. Hoaglin, and Elena Kulinskaya

Estimation in meta-analyses of mean difference and standardized mean difference

Relation of I^2 to parameters underlying our simulations

Figure C1 and C2 are produced for $q = 0.5$ and $q = 0.75$ with N=20, 40, 100, 250, $\tau^2 \in [0, 1]$ for MD and $\tau^2 = 0.5,\ 1,\ 1.5,\ 2,$ $\delta = 0,\ 0.2,\ 0.5,\ 1,\ 2$ for SMD.

The heterogeneity measure I^2 is calculated as

$$I^2 = 100\tau^2/(\tau^2 + s^2),$$

where s^2 is the average within-study variance.

In our simulations, the sample sizes for the Treatment and Control arms, $n_T = (1-q)n$ and $n_C = n-n_T$, are constant across all studies. The within-study variances, given by Equations (2.1) and (2.3), are also equal, $v_i^2 \equiv s^2$, when the true sample variances σ_{ij}^2 are substituted for their estimates s_{ij}^2 in Equation (2.1), and δ^2 is substituted for g_i in Equation (2.3).

MD: When $q = .5$ and $.75$ and $\sigma_T^2 = 1$ and 2, Figure C1 plots I^2 versus $\tau^2 \in [0, 1]$. Each panel contains traces for $n = 20, 40, 100, 250$.

SMD: When $\tau^2 = 0.5,\ 1,\ 1.5,\ 2$ and $q = .5$ and $.75$, Figure C2 plots I^2 versus δ (= 0, 0.2, 0.5, 1, 2). Each panel contains traces for $n = 20, 40, 100, 250$.

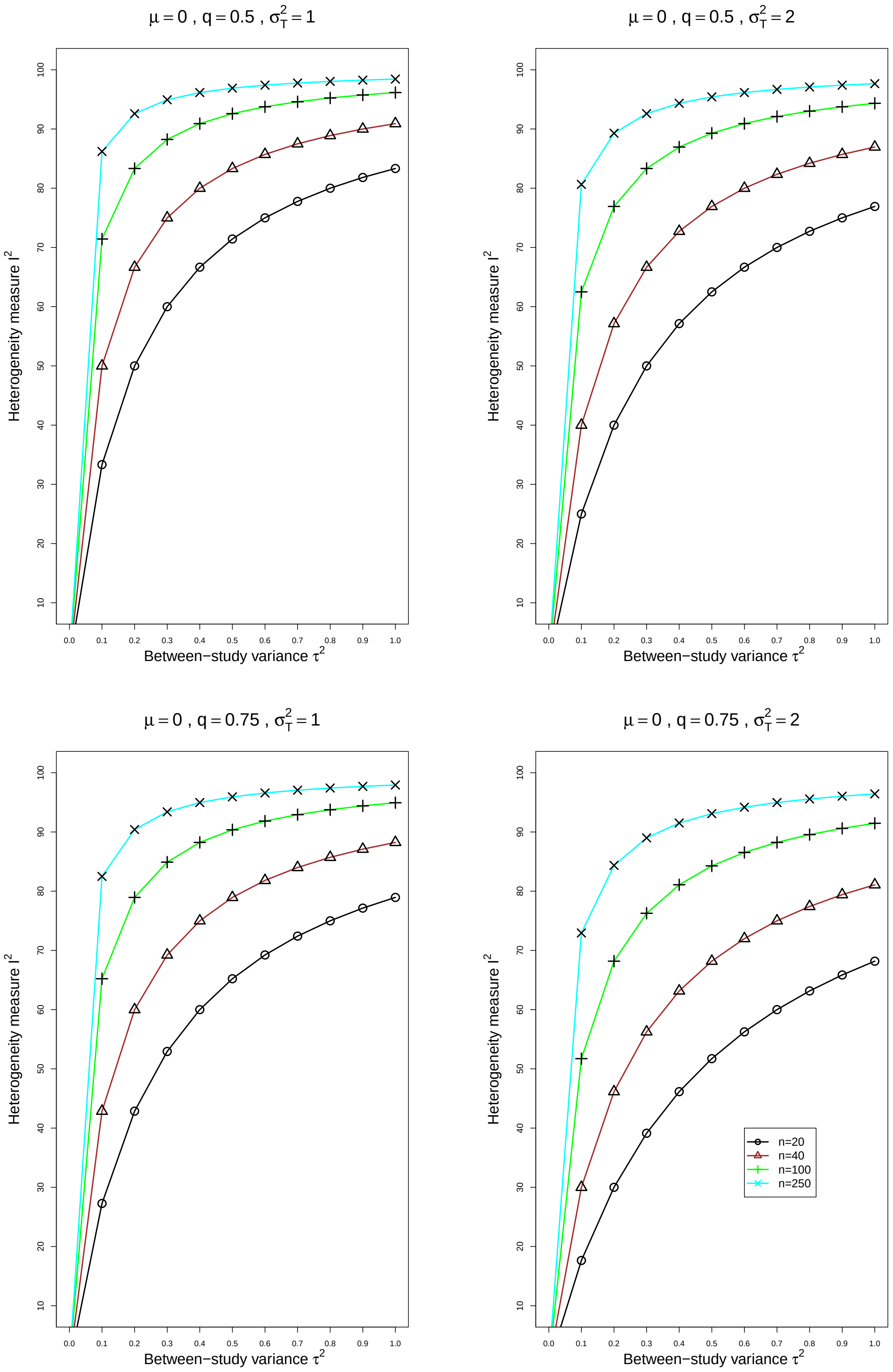

FIGURE C1 Heterogeneity measure I^2 versus $\tau^2 \in [0, 1]$ for MD when $q = .5$ and $.75$, $\sigma_T^2 = 1$ and 2, and $n = 20, 40, 100, 250$.

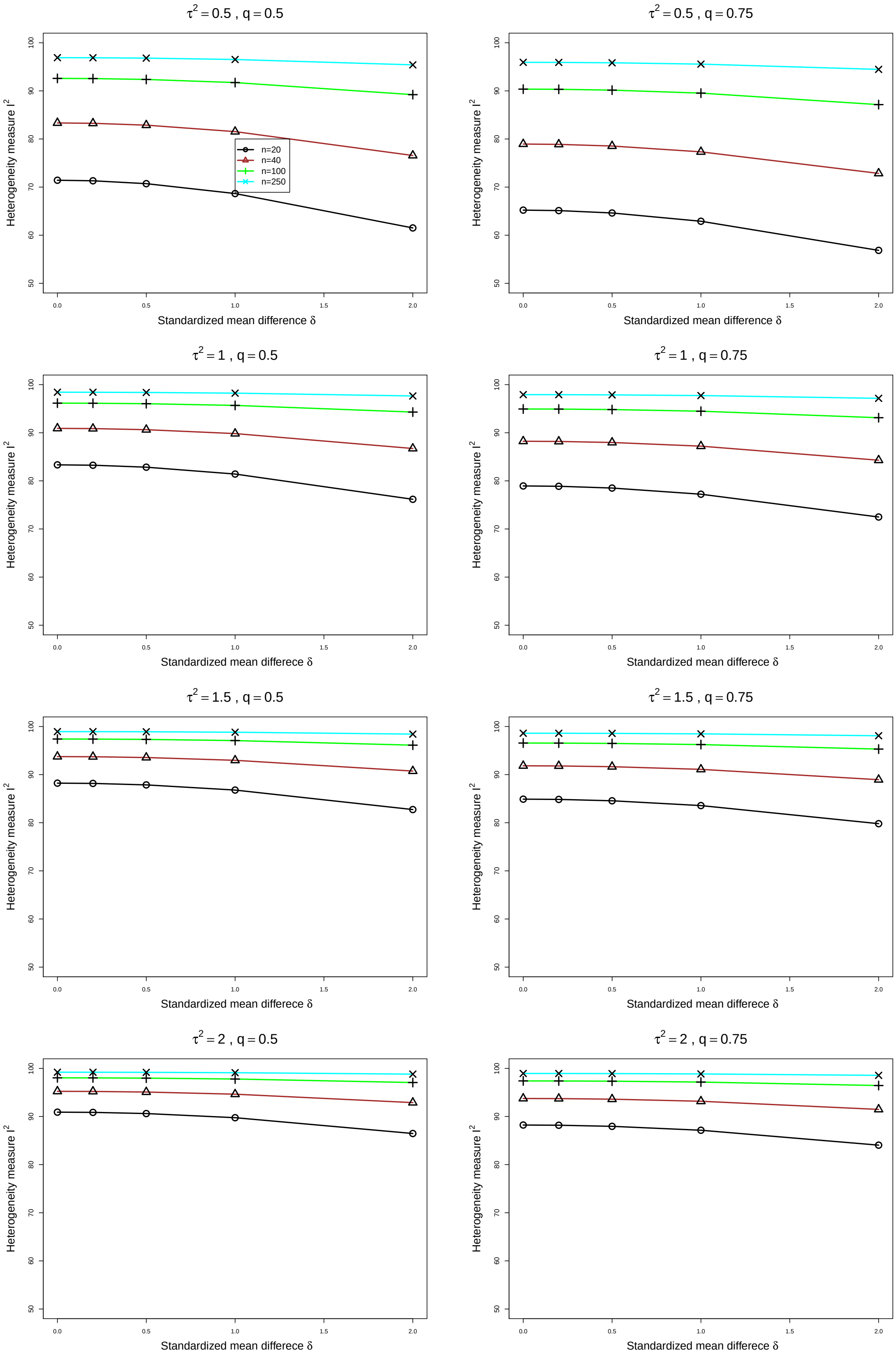

FIGURE C2 Heterogeneity measure I^2 versus standardized mean difference $\delta \in [0, 2]$ when $\tau^2 = 0.5,\ 1,\ 1.5,\ 2$; $q = .5$ and .75; and $n = 20, 40, 100, 250$.
