# Supplementary material for: Estimation in meta‐analyses of mean difference and standardized mean difference
Source: Stat Med. 2019 Nov 11;39(2):171–91. doi: 10.1002/sim.8422 (PMC6916299; doi:10.1002/sim.8422)
Supplement: Supplementary file 1 — SIM_8422‐Supp‐0001.zip [file SIM-39-171-s001.zip › MD_SMD_WebAppendix_D0.pdf]

APPENDIX

Web Appendix D

for

Ilyas Bakbergenuly, David C. Hoaglin, and Elena Kulinskaya

Estimation in meta-analyses of mean difference and standardized mean difference

DESCRIPTION OF RESULTS OF SIMULATIONS FOR MEAN DIFFERENCE (MD)

Our full simulation results, comprising 130 figures, each presenting 12 combinations of the 4 values of n or $\bar{n}$ and the 3 values of K, are provided in Appendices A and B in Bakbergenuly et al.[1]. A summary is given below.

D.1 BIAS IN ESTIMATION OF τ^2 (APPENDICES A1, A3 AND A5 IN BAKBERGENULY ET AL.[1])

All of the estimators (DL, REML, J, MP, CDL and WT) have positive bias when $\tau^2 = 0$. In favorable situations (e.g., equal n's, $q = .5$, and $\sigma_C^2 = \sigma_T^2 = 1$, as in Figure A1.1), the bias of the estimators other than WT is slightly greater than 0 when $\tau^2 = 0$, and it decreases to near 0 as $\tau^2 \to 1$ and becomes closer to 0 as n increases. In some situations, however, the bias is much greater across the range of τ^2; in the most extreme case in our simulations ($n = 20$, $K = 5$, $\sigma_C^2 = 10$, and $\sigma_T^2 = 20$; Figure A5.7), the bias at $\tau^2 = 0$ ranges from 1.6 (MP, J) to 1.8 (DL, REML) and decreases only to 1.4 to 1.7 at $\tau^2 = 1$. Generally, the bias decreases slightly as K increases. Unbalanced arms ($q = .75$) magnify the bias, especially for the smaller values of n and $\bar{n}$ and unequal variances, i.e. when $\sigma_T^2 = 2$ or $\sigma_T^2 = 20$. Among the estimators other than CDL and WT, DL and REML generally have the most bias, followed by J and MP.

The trace in the bias of CDL parallels that of DL, but it is considerably less biased than all the standard estimators. This favorable difference between CDL and the standard estimators is especially pronounced for small and unequal sample sizes. CDL is practically unbiased in the case of equal within-arm variances, and its bias is considerably less than that of DL in the most extreme cases of small and unequal sample sizes combined with unequal variances, as in Figure A5.7.

The bias of WT follows a strikingly different pattern. For small τ^2 it is positive and smaller than (or equal to) that of the other estimators. As τ^2 increases, the bias of WT becomes negative and takes increasingly more negative values, roughly linearly in τ^2. The crossover point decreases as K increases (e.g., 0.15 at $K = 5$, 0.08 at $K = 10$, and 0.05 at $K = 30$, when $n = 20$, $q = .5$, $\sigma_C^2 = 1$, and $\sigma_T^2 = 1$), but is substantially larger when σ_C^2 and σ_T^2 are large (Figures A5.1–A5.8). The slope against τ^2 flattens substantially as n (or $\bar{n}$) increases.

In summary, except for CDL and WT, the estimators of τ^2 (DL, REML, J, and MP) have non-negligible positive bias, especially for small sample sizes ($n \leq 40$) and small values of τ^2. Overall, CDL is the least biased, and WT is the least biased when the values of τ^2 are considerably smaller than the within-study variances σ_i^2 (say, when $\tau^2 \leq 0.2$ for $\sigma^2 = 1$, and when $\tau^2 \leq 1$ for $\sigma^2 = 10$). All other estimators become acceptable for larger sample sizes $n \geq 100$.

D.2 COVERAGE IN ESTIMATION OF τ^2 (APPENDICES A2, A4 AND A6 IN BAKBERGENULY ET AL.[1])

The relation between coverage of the interval estimators (PL, QP, BJ, J, and WT) and τ^2 involves other variables. When n or $\bar{n}$ is ≥ 100, most of the interval estimators have coverage close to .95 when $\tau^2 \geq 0.1$, but noticeably above .95 when $\tau^2 = 0$. (When σ_C^2 and σ_T^2 are large, PL often has coverage around .97.) When $K = 30$ and $n = 20$ (or $\bar{n} = 30$) or $n = 40$ (or $\bar{n} = 60$), QP, PL, and BJ have quite low coverage at the smallest values of τ^2 (e.g., .67 to .77 when $\tau^2 = 0$ and .83 to .88 when $\tau^2 = 0.1$, and $n = 20$, $K = 30$, $q = .75$, $\sigma_C^2 = 1$, and $\sigma_T^2 = 1$; Figures A4.3 and A2.3); the low coverage sometimes extends to most $\tau^2 \in [0, 1]$, and the departures are substantially greater for $q = .75$ than for $q = .5$. In those situations the coverage of WT is usually close to .95 and is seldom below .90. Otherwise, when $\tau^2 > 0$, the coverage of WT is usually between .95 and .96 and sometimes slightly greater. The impact of σ_C^2 and σ_T^2 (large vs. small, unequal vs. equal) is generally small.

In summary, none of the interval estimators of τ^2 (PL, QP, BJ, J, and WT) consistently achieve coverage close to .95 (i.e., between .94 and .96). All have difficulty at $\tau^2 = 0$, usually overcoverage; the departures of PL extend to other small τ^2, and its coverage is often greater than .96 but sometimes less than .94. Meta-analyses in which the studies have small sample sizes are challenging for PL, QP, BJ, and J, which in some situations have coverage well below nominal for all $\tau^2 \in [0, 1]$, especially when the number of studies is larger ($K = 30$ vs. $K = 5$ and $K = 10$). Overall, WT comes closest to providing nominal coverage of τ^2. (The contrast in behavior between the WT interval and point estimators is surprising, but the two are defined in different ways.)

D.3 BIAS IN ESTIMATION OF μ (APPENDICES B1, B3 AND B5 IN BAKBERGENULY ET AL.[1])

Because the estimated MD and its estimated variance are independent, all the estimators of μ are practically unbiased in all situations.

D.4 COVERAGE IN ESTIMATION OF μ (APPENDICES B2, B4 AND B6 IN BAKBERGENULY ET AL.[1])

When $\tau^2 \geq 0.1$ and $K \leq 10$, the methods that use critical values from the normal distribution (DL, REML, J, MP, CDL and WT) have coverage substantially below .95 (< .90 when $K = 5$ and around .92 when $K = 10$), and those coverages generally change only slightly as τ^2 increases to 1. WT is lowest, in part because it underestimates τ^2 (Figure B1.1). HKSJ and HKSJ WT have coverage close to .95. Coverage of SSW WT exceeds .95 and decreases toward .95 as τ^2 increases. The estimators other than HKSJ- and SSW-type have coverage slightly > .95 when $\tau^2 = 0$. HKSJ and HKSJ WT are around .94 when $\tau^2 = 0$, and SSW WT is > .99 when $K = 5$, decreasing to around .965 when $K = 30$. For $0 < \tau^2 < 0.1$, all estimators other than SSW-type have coverage below nominal for small sample sizes, considerably below for unequal sample sizes $\bar{n} \leq 60$ and $K \leq 10$ (Appendix B4).

When the studies' n's are equal, coverage of most profile-type estimators *decreases* as n increases; this pattern is absent when the n's are unequal. The coverage of WT increases to the level of the other profile-type estimators.

As K increases, the coverage of the profile-type estimators approaches .95 (from below).

When $n = 20$ or $\bar{n} = 30$, the traces for the various estimators show more separation when $q = .75$ than when $q = .5$. This pattern is most noticeable when the σ^2's are large and $K = 30$. It is in these circumstances that SSW CDL is considerably better than SSW WT, achieving nominal coverage for larger values of τ^2 or K, as in Figure B2.7.

Undercoverage is somewhat less when $\sigma_C^2 = \sigma_T^2 = 10$ than when $\sigma_C^2 = \sigma_T^2 = 1$. Coverage differs little between $\sigma_T^2 = 2$ and $\sigma_T^2 = 1$ and between $\sigma_T^2 = 20$ and $\sigma_T^2 = 10$.

In summary, HKSJ and HKSJ WT generally (but not uniformly) have the best coverage. Their coverage is not always within $\pm.01$ of .95; it may be considerably below nominal for $\tau^2 < 0.1$ when sample sizes are small; but in situations where clear differences separate the interval estimators, HKSJ and HKSJ WT are much closer to .95. DL, WT, MP, REML, and J exhibit very serious undercoverage when $K = 5$ and nontrivial undercoverage when $K = 10$. For small and/or unbalanced sample sizes, SSW CDL is the only estimator achieving nominal coverage for larger values of τ^2 or K.

References

1. Bakbergenuly I, Hoaglin DC, Kulinskaya E. Simulation study of estimating between-study variance and overall effect in meta-analysis of mean difference. *eprint arXiv:1904.01948v1 [stat.ME]* 2019.
