# Supplementary material for: Estimation in meta‐analyses of mean difference and standardized mean difference
Source: Stat Med. 2019 Nov 11;39(2):171–91. doi: 10.1002/sim.8422 (PMC6916299; doi:10.1002/sim.8422)
Supplement: Supplementary file 1 — SIM_8422‐Supp‐0001.zip [file SIM-39-171-s001.zip › MD_SMD_WebAppendix_E0.pdf]

APPENDIX

Web Appendix E

for

Ilyas Bakbergenuly, David C. Hoaglin, and Elena Kulinskaya

Estimation in meta-analyses of mean difference and standardized mean difference

DESCRIPTION OF RESULTS OF SIMULATIONS FOR STANDARDIZED MEAN DIFFERENCE (SMD)

Our full simulation results, comprising 130 figures, each presenting 12 combinations of the 4 values of n or $\bar{n}$ and the 3 values of K, are provided in Appendices A and B in in Bakbergenuly et al.[1]. A summary is given below.

E.1 BIAS IN ESTIMATION OF τ^2 (APPENDIX A1 IN BAKBERGENULY ET AL.[1])

The five estimators (DL, REML, J, MP, and KDB) have biases whose traces fan out from the same small positive bias at $\tau^2 = 0$. As τ^2 increases, KDB remains positive and increases slowly; MP stays close to 0 (and slightly below); REML stays negative, with a negative slope; J stays negative, with a more-negative slope; and DL becomes increasingly negative, showing noticeable curvature. The value of δ has little effect on this pattern (except that the bias of DL and J has smaller magnitude when $\delta = 2$).

As n or $\bar{n}$ increases, the traces for KDB, MP, and REML flatten, and their bias is essentially 0 when n or $\bar{n} = 100$. The traces for J and DL become less steep, but substantial bias remains at $n = 250$ (or $\bar{n} = 160$).

As K increases, the trace for KDB flattens somewhat, but the traces for the other estimators become steeper.

The traces for J and DL are slightly less steep when $q = .75$ than when $q = .5$.

In summary, the patterns of bias indicate a choice among the five estimators of τ^2 (DL, REML, J, MP, and KDB). When $n \leq 40$, MP is closer to unbiased than KDB when $K = 5$, the magnitudes of their biases are roughly equal when $K = 10$, and KDB is closer to unbiased when $K = 30$. When $n \geq 100$, MP, KDB, and REML are nearly unbiased. DL and J seriously underestimate τ^2. The average of MP and KDB should be close to unbiased.

E.2 COVERAGE IN ESTIMATION OF τ^2 (APPENDIX A2 IN BAKBERGENULY ET AL.[1])

The five estimators (PL, QP, BJ, J, and KDB) share the feature that their coverage decreases as τ^2 increases from 0 to 0.5. At $\tau^2 = 0$ all five have coverage $\geq .95$. KDB is highest (e.g., .99 when $q = .5$ and $n = 20$), but it drops below .95 at $\tau^2 = 0.5$ or $\tau^2 = 1.0$ and remains slightly below .95. BJ is next highest (e.g., .98 when $q = .5$ and $n = 20$), and it remains above .95 (say, .96 to .97) when $K = 5$ and $K = 10$. QP is close to .95 for $\tau^2 \geq 0.5$. PL is between BJ and QP, and it remains above QP when $K = 30$. The trace for BJ behaves quite differently when $K = 30$ than when $K \leq 10$, decreasing steeply and linearly to around .77 at $\tau^2 = 2.5$ (when $q = .5$ and $n = 20$). When $K \leq 10$, J is between BJ and QP; and when $K = 30$, it also decreases linearly, but less steeply (e.g., to around .92 at $\tau^2 = 2.5$ when $q = .5$ and $n = 20$).

Coverage does not change noticeably as n or $\bar{n}$ increases, when $K \leq 10$. When $K = 30$, the slopes of BJ and J become slightly less steep, and the traces of the other estimators move closer together and are closer to .95.

Setting aside the behavior of BJ and J when $K = 30$, and of the other estimators when $n = 20$ and $K = 30$, the traces move closer together as K increases.

The slopes of BJ and J when $K = 30$ are less steep when $q = .75$ than when $q = .5$.

As δ increases, the coverage of QP at $K = 30$ increases slightly; it is substantially closer to .95 when $\delta = 2$.

In summary, all five interval estimators of τ^2 have coverage substantially above .95 when $\tau^2 = 0$. When $\tau^2 \geq 0.5$, QP is generally closest to .95. The unusual behavior of BJ (and, to a lesser extent, J) when $K = 30$ adds to the evidence against it.

E.3 BIAS AND MEAN SQUARED ERROR IN ESTIMATION OF δ (APPENDIX B1 IN BAKBERGENULY ET AL.[1])

When $\delta = 0$, the bias of the six estimators (DL, REML, MP, KDB, J, and SSW) follows a single trace, close to 0, for all values of τ^2. When $\delta > 0$, SSW stays close to 0, and the others shift down, to increasingly negative bias, as δ increases, and their traces separate. For example, when $\delta = 1$, $q = .5$, $n = 20$, $K = 10$, and $\tau^2 = 2.5$, the bias ranges from -0.05 (KDB) to -0.07 (DL), and MP, REML, and J (in that order) have intermediate values.

When $\delta \leq 0.5$, bias has little relation to τ^2. When $\delta \geq 1$, however, the bias of the estimators other than SSW (especially DL) becomes increasingly negative as τ^2 increases. (The plot for $n = 20$ and $K = 30$ in B1.17 shows an extreme example.)

Where bias is nonzero, increasing n or $\bar{n}$ moves the traces toward (or to) 0, decreasing separation between them. Some plots (e.g., B1.37 and B1.39) show slight evidence of greater separation among traces when sample sizes are unequal and $\delta = 2$.

For the most part, K has little or no effect on bias. Some plots suggest that, where bias is nonzero, separation among traces increases as K increases, especially from $K = 10$ to $K = 30$.

Bias does not differ noticeably between $q = .5$ and $q = .75$.

SSW essentially avoids the bias that we found in the inverse-variance-weighted estimators of δ. To provide an additional measure of its performance (besides coverage, discussed below). we estimated the mean squared error of SSW and the

best two inverse-variance-weighted estimators, KDB and MP. Appendix E1 includes figures that plot (versus τ^2) the ratios MSE(SSW)/MSE(KDB) and MSE(SSW)/MSE(MP) for the five values of δ, the two values of q, and $n_i = 20,\ 40,\ 100,\ 250$. For most situations the two ratios are essentially equal and differ little among values of K and q. In most situations the traces are essentially flat as τ^2 increases; otherwise, they curve downward as τ^2 approaches 0. As n increases, the ratios approach 1. For example, when $\delta = 0$ and $K = 5$, they decrease from around 1.1 when $n = 20$ to nearly 1.0 when $n = 250$. As δ increases (≥ 0.5), the ratios at small τ^2 decrease. This pattern is first noticeable when $\delta = 0.5$ and $n = 20$ and $K = 30$; and as δ increases, it becomes more pronounced at that combination of n and K and extends to larger n (with $K = 30$) and to $n \leq 40$ and $K = 10$. When $\delta = 2$, $q = .5$, $n = 20$, and $K = 30$, the traces for the two ratios are separate and curve up from around 0.55 at $\tau^2 = 0$ to slightly < 1 when $\tau^2 = 2.5$. The patterns are similar for $q = .75$.

In summary, the bias of SSW is close to 0, and the other five estimators (DL, REML, J, MP, and KDB), which use inverse-variance weights, have greater (and negative) bias, amounting to 5 – 10% when sample sizes are small and $\delta \geq 1$. This bias increases as τ^2 increases. SSW usually has slightly greater mean squared error than KDB and MP when n is small, but its MSE can be substantially smaller, especially for small τ^2.

E.4 COVERAGE IN ESTIMATION OF δ (APPENDIX B2 IN BAKBERGENULY ET AL.[1])

Coverage of the estimators that rely on inverse-variance weights and normal critical values (DL, REML, MP, KDB, and J) is influenced most by τ^2 (= 0 versus > 0) and K. At $\tau^2 = 0$ their coverage is around .97, but at $\tau^2 = 0.5$ (when $q = .5$ and $n = 20$) it is mostly below .95: .90 to .91 when $K = 5$, .92 to.94 when $K = 10$, and .93 to .95+ when $K = 30$. As τ^2 increases, their coverage either is flat (REML, MP, and KDB) or decreases (DL, J). DL almost always has the lowest coverage, and the gap between it and J widens as K increases.

Except for the effect of K on SSW KDB at $\tau^2 = 0$ (above .99 when $K = 5$, decreasing to .97 when $K = 30$) and below-nominal coverage of HKSJ and HKSJ KDB in a region whose definition involves mainly δ, n (or $\bar{n}$), K, and τ^2, the coverage of SSW and the HKSJ-type estimators is close to .95 for all K and τ^2. The challenging situations in that region generally involve $\delta = 1$ or $\delta = 2$, the smaller n or $\bar{n}$, $K = 10$ or $K = 30$, and the smaller τ^2. For example, when $\delta = 2$ and $n = 20$ or $\bar{n} = 30$ and $K = 30$, coverage can be as low as .84 when $\tau^2 = 0$.

When $\tau^2 \geq 0.5$, coverage of DL, REML, MP, KDB, and J increases as K increases, usually staying below .95.

The effect of δ on coverage is slight except for some situations involving $\delta = 2$. When $n = 20$ and $K = 30$, all of the estimators except SSW KDB have low coverage at $\tau^2 = 0$, ranging from < .82 to .86. Their traces rise as τ^2 increases; when $\tau^2 = 2.5$, KDB and HKSJ KDB are almost .94, and DL is .86 (up from .84). The pattern is similar when $n = 40$ and $K = 30$, but much reduced.

When $\tau^2 \geq 0.5$, coverage *decreases* slightly as n or $\bar{n}$ increases (except for SSW and the two HKSJ-type estimators); coverage is somewhat lower when sample sizes are unequal.

The plots show at most slight differences between $q = .5$ and $q = .75$.

In summary, except when $\delta = 2$ and $K = 30$, HKSJ and HKSJ KDB have coverage closest to .95; they differ little, and departures from .95 (toward lower coverage) are seldom serious. SSW KDB is rather conservative when $K = 5$ and for other K when $\tau^2 = 0$. Otherwise it provides reliable, albeit slightly conservative, coverage. When $\delta = 2$ and $K = 30$, SSW KDB is the best alternative. All of the estimators that use inverse-variance weights and critical values from the normal distribution (DL,REML, J, MP, and KDB) often have coverage substantially below .95.

References

1. Bakbergenuly I, Hoaglin DC, Kulinskaya E. Simulation study of estimating between-study variance and overall effect in meta-analysis of standardized mean difference. *eprint arXiv:1903.01362v1 [stat.ME]* 2019.
