# Supplementary material for: Estimation in meta‐analyses of mean difference and standardized mean difference
Source: Stat Med. 2019 Nov 11;39(2):171–91. doi: 10.1002/sim.8422 (PMC6916299; doi:10.1002/sim.8422)
Supplement: Supplementary file 1 — SIM_8422‐Supp‐0001.zip [file SIM-39-171-s001.zip › MD_SMD_WebAppendix_F0.pdf]

APPENDIX

Web Appendix F

for

Ilyas Bakbergenuly, David C. Hoaglin, and Elena Kulinskaya

Estimation in meta-analyses of mean difference and standardized mean difference

F1 R PROCEDURES TO IMPLEMENT WT, CDL AND SSW ANALYSES WITH MEAN DIFFERENCES

F1.1 Example - studies on the effectiveness of glucose-lowering agents using WT, SSW and HKSJ

```
#R version 3.4.2 (2017-09-28)
#Senn, S., Gavini, F., Magrez, D., & Scheen, A. (2013).
#Issues in performing a #network meta-analysis.
#Statistical Methods in Medical Research, 22, 169-189.
#Data extraction for meta-analysis
library(metafor)
dat <- get(data(dat.senn2013))
dat <- dat.senn2013[,c(1,4,3,2,5,6)]
dat.c <- lapply(split(dat, dat$study),
function(x) cbind(x[rep(1,nrow(x)-1),], x[-1,c(3:6)]))
dat.c <- do.call(rbind, dat.c)
names(dat.c)[3:10] <- c("trt1", "n1i", "m1i", "sd1i", "trt2", "n2i", "m2i", "sd2i")
rownames(dat.c) <- 1:nrow(dat.c)
dat.c$id <- 1:nrow(dat.c)
dat.c
dat.c <- escalc(measure="MD", m1i=m1i, sd1i=sd1i, n1i=n1i, m2i=m2i, sd2i=sd2i, n2i=
    n2i, data=dat.c)
yi<-as.numeric(dat.c$yi)
vi<-as.numeric(dat.c$vi)
nT<-dat.c$n1i
```

```
nC<-dat.c$n2i
sT<-dat.c$sd1i
sC<-dat.c$sd2i
##################################################
res1<-metaMDWelch(yi,vi,nC,nT,sC,sT)
#             estimate      ci.lb      ci.ub
#tau2 (WT) 0.2453677 0.1390765 0.4946575
res2<-metaSSWwithWT(yi,vi,nC,nT,sC,sT)
#                     estimate       ci.lb       ci.ub
#tau2 (WT)            0.2453677   0.1390765   0.4946575
#theta (SSW WT) -0.8372593 -1.0835101 -0.5910084
res3<-metaHKSJwithWT(yi,vi,nC,nT,sC,sT)
#                      estimate       ci.lb       ci.ub
#tau2 (WT)             0.2453677   0.1390765   0.4946575
#theta (HKSJ WT) -0.7907464 -1.0033276 -0.5781652
```

F1.2 Example - studies on the effectiveness of glucose-lowering agents using CDL, SSW and HKSJ

```
#R version 3.4.2 (2017-09-28)
#Senn, S., Gavini, F., Magrez, D., & Scheen, A. (2013).
#Issues in performing a #network meta-analysis.
#Statistical Methods in Medical Research, 22, 169-189.
#Data extraction for meta-analysis
library(metafor)
dat <- get(data(dat.senn2013))
dat <- dat.senn2013[,c(1,4,3,2,5,6)]
dat.c <- lapply(split(dat, dat$study),
function(x) cbind(x[rep(1,nrow(x)-1),], x[-1,c(3:6)]))
dat.c <- do.call(rbind, dat.c)
names(dat.c)[3:10] <- c("trt1", "n1i", "m1i", "sd1i", "trt2", "n2i", "m2i", "sd2i")
rownames(dat.c) <- 1:nrow(dat.c)
dat.c$id <- 1:nrow(dat.c)
dat.c
dat.c <- escalc(measure="MD", m1i=m1i, sd1i=sd1i, n1i=n1i, m2i=m2i, sd2i=sd2i, n2i=
    n2i, data=dat.c)
```

```
yi<-as.numeric(dat.c$yi)
vi<-as.numeric(dat.c$vi)
nT<-dat.c$n1i
nC<-dat.c$n2i
sT<-dat.c$sd1i
sC<-dat.c$sd2i
##################################################
res4<-metaMDwithCDL(yi,vi,nC,nT)
#              estimate      ci.lb      ci.ub
#tau2 (CDL)  0.2822965  0.1390765  0.4946575
res5<-metaSSWwithCDL(yi,vi,nC,nT)
#                      estimate       ci.lb       ci.ub
#tau2 (CDL)           0.2822965   0.1390765   0.4946575
#theta (SSW CDL)     -0.8372593  -1.1003075  -0.5742110
res6<-metaHKSJwithCDL(yi,vi,nC,nT)
#                      estimate       ci.lb       ci.ub
#tau2 (CDL)           0.2822965   0.1390765   0.4946575
#theta (HKSJ CDL)    -0.7891808  -1.0017326  -0.5766290
```

F1.3 Meta-analysis using WT and SSW

F1.3.1 Function - metaSSWwithWT

```
#Program for Mean Differences
#User needs to input values for effects yi and within-study variances vi#
#and also sample sizes nC and nT in control and treatment group#####
metaSSWwithWT <- function(yi,vi,nC,nT,sC,sT){
        #estimation of between study variance
        tau2_estimates <- metaMDWelch(yi,vi,nC,nT,sC,sT)
        tau2 <- tau2_estimates[[1]]
        K <- length(yi)
        n=nC+nT
        nbar <- (nC*nT)/n
        thetabar_SSW <- sum(yi*nbar)/sum(nbar)
        varThetaBar_SSW <- sum((vi+tau2)*(nbar^2))/((sum(nbar))^2)
        ######################################################################
        Ltheta_SSW <- thetabar_SSW-qt(.975, df=K-1)*sqrt(varThetaBar_SSW)
        Utheta_SSW <- thetabar_SSW+qt(.975, df=K-1)*sqrt(varThetaBar_SSW)
        theta_estimates <- data.frame(thetabar_SSW, Ltheta_SSW, Utheta_SSW)
        names(theta_estimates) <- names(tau2_estimates)
        output <- rbind(tau2_estimates,theta_estimates)
        colnames(output) <- c("estimate","ci.lb","ci.ub")
        rownames(output) <- c("tau2_(WT)","theta_(SSW_WT)")
return(output)
}
```

F1.3.2 Function - metaHKSJwithWT

```
###############Hartung Knapp Sidik Jonkman (HKSJ) method#################
metaHKSJwithWT <- function(yi,vi,nC,nT,sC,sT){
        #estimation of between study variance
        tau2_estimates <- metaMDWelch(yi,vi,nC,nT,sC,sT)
        tau2 <- tau2_estimates[[1]]
        K <- length(yi)
        thetabar_HKSJ <- sum(yi/(vi+tau2))/sum(1/(vi+tau2))
        w_HKSJ <- 1/(vi+tau2)
```

```
        var_HKSJ <- sum(w_HKSJ*(yi-thetabar_HKSJ)^2)/((K-1)*sum(w_HKSJ))
        Ltheta_HKSJ <- thetabar_HKSJ-qt(.975, df=K-1)*sqrt(var_HKSJ)
        Utheta_HKSJ <- thetabar_HKSJ+qt(.975, df=K-1)*sqrt(var_HKSJ)
        theta_estimates <- data.frame(thetabar_HKSJ,Ltheta_HKSJ,Utheta_HKSJ)
        names(theta_estimates) <- names(tau2_estimates)
        output <- rbind(tau2_estimates,theta_estimates)
        colnames(output) <- c("estimate","ci.lb","ci.ub")
        rownames(output) <- c("tau2_(WT)","theta_(HKSJ_WT)")
return(output)
}
```

F1.3.3 Function - metaMDWelch

```
########################################################################
#######Mandel-Paule method based on Welch-type  approximation##########
########################################################################
##required functions:ImprovedQmomentsMD
metaMDWelch<-function(yi,vi,nC,nT,sC,sT){
        ##initial parameters
        ll <- 0.000001
        uu <- 1000
        parameters <- ImprovedQmomentsMD(yi,vi,nC,nT,sC,sT)
        EQ <- parameters[[1]]
        df1 <- parameters[[2]]
        df2 <- parameters[[3]]
        C <- parameters[[4]]
        ####################
        #initialize point and interval estimates of between study variance
        tau2.lb_WT <- 0
        tau2.ub_WT <- 0
        tau2_WT <- 0
        #improved first moment of Q
        f_WT <- function(g,sigma,theta,EQ){
        sum((theta-sum(theta/(sigma+g))/sum(1/(sigma+g)))^2/(sigma+g))-EQ}
        #function for calculation of point estimate of between study variance
```

```
    if (f_WT(ll,sigma=vi,theta=yi,EQ=EQ)*f_WT(uu,sigma=vi,theta=yi,EQ=EQ)<0)
    {
    tau2_WT <-  as.numeric(uniroot(f_WT, c(ll, uu), tol = 0.0001, sigma=vi,theta
        =yi,EQ=EQ)[1])}
    #function for calculation of upper bound
    f_upper_WT <- function(g,sigma,theta,C,df1,df2){
    sum((theta-sum(theta/(sigma+g))/sum(1/(sigma+g)))^2/(sigma+g))-C*qf(0.025,
        df1, df2)}
    #calculation of upper bound
    if (f_upper_WT(ll,sigma=vi,theta=yi,C=C,df1=df1,df2=df2)*f_upper_WT(uu,sigma
        =vi,theta=yi,C=C,df1=df1,df2=df2)<0)
    {
    tau2.ub_WT <-  as.numeric(uniroot(f_upper_WT, c(ll, uu), tol = 0.0001, sigma
        =vi,theta=yi,C=C,df1=df1,df2=df2)[1])}
    #function for calculation of lower bound
    f_lower_WT <- function(g,sigma,theta,C,df1,df2){
    sum((theta-sum(theta/(sigma+g))/sum(1/(sigma+g)))^2/(sigma+g))-C*qf(0.975,
        df1, df2)}
    #calculation of lower bound
    if (f_lower_WT(ll,sigma=vi,theta=yi,C=C,df1=df1,df2=df2)*f_lower_WT(uu,sigma
        =vi,theta=yi,C=C,df1=df1,df2=df2)<0)
    {
    tau2.lb_WT <- as.numeric(uniroot(f_lower_WT, c(ll, uu), tol = 0.0001, sigma=
        vi,theta=yi,C=C,df1=df1,df2=df2)[1])}
    tau2=data.frame(tau2_WT,tau2.lb_WT,tau2.ub_WT)
    colnames(tau2)  <-  c("estimate", "ci.lb", "ci.ub")
    rownames(tau2)  <-  c("tau2_(WT)")
    return(tau2)
}
```

F1.3.4 Function - ImprovedQmomentsMD

```
ImprovedQmomentsMD <- function(yi, vi, nC, nT, sC, sT){
        #inverse-variance weights
        w <- (vi)^(-1)
        ##total sample size
        N <- nT+nC
        ###Corrected Mandel-Paule method based on
        ###Welch distribution proposed in a paper
        ###by Kulinskaya E, Dollinger M, Knight E, Gao H.A,
        ###Statistics in Medicine, (2004)
        ###A Welch-type test for homogeneity of contrasts
        ###under heteroscedasticity with application to meta-analysis.
        K <- length(N)
        W <- sum(w)
        x <- yi
        xbar <- sum(x*w)/W
        p <- 1-w/W
        g <- sC^4/(nC^2*(nC-1))+sT^4/(nT^2*(nT-1))
        M1 <- K-1+2*sum(w^2*g*p^2)
        M2 <- 2*(K-1)+14*sum(w^2*g*p^2)
        f2 <- (2*(K-3)*M1^2+4*(K-1)*M2)/((K-1)*M2-2*M1^2)
        c <- M1*(f2-2)/f2
        ##parameters
        EQ <- M1
        df1 <- (K-1)
        df2 <- f2
        C <- c
        moments=data.frame(EQ, df1, df2, C)
        colnames(moments) <- c("EQ", "df1", "df2", "C")
        return(moments)
}
```

F1.4 Meta-analysis using CDL and SSW

F1.4.1 Function - metaSSWwithCDL

```
metaSSWwithCDL <- function(yi,vi,nC,nT){
        #estimation of between study variance
        tau2_estimates <- metaMDwithCDL(yi,vi,nC,nT)
        tau2 <- tau2_estimates[[1]]
        K <- length(yi)
        n=nC+nT
        nbar <- (nC*nT)/n
        thetabar_SSW <- sum(yi*nbar)/sum(nbar)
        varThetaBar_SSW <- sum((vi+tau2)*(nbar^2))/((sum(nbar))^2)
        ##########################################################################
        Ltheta_SSW <- thetabar_SSW-qt(.975, df=K-1)*sqrt(varThetaBar_SSW)
        Utheta_SSW <- thetabar_SSW+qt(.975, df=K-1)*sqrt(varThetaBar_SSW)
        theta_estimates <- data.frame(thetabar_SSW, Ltheta_SSW, Utheta_SSW)
        names(theta_estimates) <- names(tau2_estimates)
        output <- rbind(tau2_estimates,theta_estimates)
        colnames(output) <- c("estimate","ci.lb","ci.ub")
        rownames(output) <- c("tau2_(CDL)","theta_(SSW_CDL)")
return(output)
}
```

F1.4.2 Function - metaHKSJwithCDL

```
###############Hartung Knapp Sidik Jonkman (HKSJ) method#################
metaHKSJwithCDL <- function(yi,vi,nC,nT){
        #estimation of between study variance
        tau2_estimates <- metaMDwithCDL(yi,vi,nC,nT)
        tau2 <- tau2_estimates[[1]]
        K <- length(yi)
        thetabar_HKSJ <- sum(yi/(vi+tau2))/sum(1/(vi+tau2))
        w_HKSJ <- 1/(vi+tau2)
        var_HKSJ <- sum(w_HKSJ*(yi-thetabar_HKSJ)^2)/((K-1)*sum(w_HKSJ))
        Ltheta_HKSJ <- thetabar_HKSJ-qt(.975, df=K-1)*sqrt(var_HKSJ)
        Utheta_HKSJ <- thetabar_HKSJ+qt(.975, df=K-1)*sqrt(var_HKSJ)
```

```
        theta_estimates <- data.frame(thetabar_HKSJ, Ltheta_HKSJ, Utheta_HKSJ)
        names(theta_estimates) <- names(tau2_estimates)
        output <- rbind(tau2_estimates, theta_estimates)
        colnames(output) <- c("estimate","ci.lb","ci.ub")
        rownames(output) <- c("tau2_(CDL)","theta_(HKSJ_CDL)")
return(output)
}
```

F1.5 Function - metaMDwithCDL

```
metaMDwithCDL<-function(yi, vi, nC, nT){
        ##initial parameters
        ll <- 0.000001
        uu <- 1000
        ##########################################
        N<-nT+nC
        K<-length(N)
        #DerSimonian and Laird method
        w<-1/vi
        W<-sum(w)
        thetabar_FE<-sum(yi*w)/W
        Q<-sum(w*(yi-thetabar_FE)^2)
        W2<-sum(w^2)
        tauDL<-max(0, (Q-K+1)/(W-W2/W))
        ##########################################
        ##Corrected DerSimonian and Laird method##
        x<-yi
        xbar<-sum(x*w)/W
        p<-1-w/W
        g<-sC^2/(nC^2*(nC-1))+sT^2/(nT^2*(nT-1))
        A=sum(w^2*g*p^2)
        B=sum(w^3*g*p*(1-2*w/W+W2/(W^2)))
        tau2.CDL<-max((Q-(K-1)-2*A)/(W-W2/W),0)
        ##########################################
        xbar <- sum(x*w)/W
```

```
p <- 1-w/W
g <- sC^4/(nC^2*(nC-1))+sT^4/(nT^2*(nT-1))
M1 <- K-1+2*sum(w^2*g*p^2)
M2 <- 2*(K-1)+14*sum(w^2*g*p^2)
f2 <- (2*(K-3)*M1^2+4*(K-1)*M2)/((K-1)*M2-2*M1^2)
C <- M1*(f2-2)/f2
##parameters
df1 <- (K-1)
df2 <- f2
############################################
#function for calculation of upper bound
f_upper_WT <- function(g,sigma,theta,C,df1,df2){
sum((theta-sum(theta/(sigma+g))/sum(1/(sigma+g)))^2/(sigma+g))-C*qf(0.025,
    df1, df2)}
#calculation of upper bound
if (f_upper_WT(ll,sigma=vi,theta=yi,C=C,df1=df1,df2=df2)*f_upper_WT(uu,sigma
    =vi,theta=yi,C=C,df1=df1,df2=df2)<0)
{
tau2.ub_WT <- as.numeric(uniroot(f_upper_WT, c(ll, uu), tol = 0.0001, sigma
    =vi,theta=yi,C=C,df1=df1,df2=df2)[1])}
#function for calculation of lower bound
f_lower_WT <- function(g,sigma,theta,C,df1,df2){
sum((theta-sum(theta/(sigma+g))/sum(1/(sigma+g)))^2/(sigma+g))-C*qf(0.975,
    df1, df2)}
#calculation of lower bound
if (f_lower_WT(ll,sigma=vi,theta=yi,C=C,df1=df1,df2=df2)*f_lower_WT(uu,sigma
    =vi,theta=yi,C=C,df1=df1,df2=df2)<0)
{
tau2.lb_WT <- as.numeric(uniroot(f_lower_WT, c(ll, uu), tol = 0.0001, sigma=
    vi,theta=yi,C=C,df1=df1,df2=df2)[1])}
tau2_estimates=data.frame(tau2.CDL, tau2.lb_WT, tau2.ub_WT)
colnames(tau2_estimates) <- c("estimate", "ci.lb", "ci.ub")
rownames(tau2_estimates) <- c("tau2_(CDL)")
return(tau2_estimates)}
```
