# Supplementary material for: Estimation in meta‐analyses of mean difference and standardized mean difference
Source: Stat Med. 2019 Nov 11;39(2):171–91. doi: 10.1002/sim.8422 (PMC6916299; doi:10.1002/sim.8422)
Supplement: Supplementary file 1 — SIM_8422‐Supp‐0001.zip [file SIM-39-171-s001.zip › MD_SMD_WebAppendix_G0.pdf]

APPENDIX

Web Appendix G

for

Ilyas Bakbergenuly, David C. Hoaglin, and Elena Kulinskaya

Estimation in meta-analyses of mean difference and standardized mean difference

G1 R PROCEDURES TO IMPLEMENT KDB AND SSW ANALYSES WITH STANDARDIZED MEAN DIFFERENCES

G1.1 Example - Efficacy of psychological treatments for obsessive-compulsive disorder

```
nT<-c(10,22,29,29,3,21,15,55,55,11,13,
9,6,10,18,16,10,6,10,11,19,19,6,10)
nC<-c(8,23,32,32,11,20,14,66,66,10,16,
9,6,10,33,33,8,6,10,15,16,16,9,9)
yi<-c(1.425,1.068,0.924,0.909,0.281,1.646,1.007,0.996,
0.731,1.882,1.082,2.326,-0.229,0.191,0.980,1.620,
2.997,0.860,1.494,0.597,0.674,0.490,3.780,1.590)
vi<-c(0.2814,0.1016,0.0727,0.0725,0.1355,0.1307,0.1556,0.0374,
0.0355, 0.2752, 0.1596,0.3725,0.3355,0.2009,0.0953,0.1196,0.4745,
0.3642,0.2558,0.1644,0.1216,0.1186,0.7541,0.2776)
#estimation of between study variance using KDB method
#for standardized mean difference
res1<-metaSMDimprovedQ(yi,vi,nC,nT)
#                 estimate    ci.lb     ci.ub
#tau2 (KDB) 0.4335939 0.1242509 1.282221
########################################################
#meta-analysis of standardized mean difference by SSW KDB method
########################################################
res2=metaSSWwithKDB(yi,vi,nC,nT)
#                        estimate     ci.lb     ci.ub
```

```
# tau2 (KDB)        0.4335939 0.1242509 1.282221
# theta (SSW KDB) 1.0950318 0.7078233 1.482240
#meta-analysis of standardized mean difference by HKSJ KDB method
#######################################################
res3<-metaHKSJwithKDB(yi, vi, nC, nT)
#                           estimate       ci.lb      ci.ub
# tau2 (KDB)          0.4335939 0.1242509 1.282221
# theta (HKSJ KDB) 1.1198009 0.8014624 1.438139
```

G1.2 Function - metaSMDimprovedQ

```
##############################################################################
#Program for Standardized Mean Differences with improved estimation of
#between study variance based on paper by
#Kulinskaya E, Dollinger M, Bjorkestol K Biometrics 2011
#User needs to input values for effects yi and within−study variances vi
#and also sample sizes nC and nT in control and treatment group
##################################################################################
#required functions:ImprovedQmomentsSMD, parm, Qdelta ,EQ,mom,cmom,EQ2
metaSMDimprovedQ<−function(yi,vi,nC,nT){
        ##initial parameters
        ll <− 0.000001
        uu <− 1000
        #initialize point and interval estimates of between study variance
        tau2.lb_KDB <− 0
        tau2.ub_KDB <− 0
        tau2_KDB <− 0
        parameters <− ImprovedQmomentsSMD(yi,vi,nC,nT)
        #improved first moment of Q
        EhatQ <− parameters[[1]]
        #function for calculation of point estimate of between study variance
        f_KDB <− function(g,sigma2,theta,EhatQ){
        sum((theta−sum(theta/(sigma2+g))/sum(1/(sigma2+g)))^2/(sigma2+g))−EhatQ}
        #point estimate of between study variance
        if(f_KDB(ll,sigma2=vi,theta=yi,EhatQ=EhatQ)*
        f_KDB(uu,sigma2=vi,theta=yi,EhatQ=EhatQ)<0){
        tau2_KDB <− as.numeric(uniroot(f_KDB, c(ll, uu), tol = 0.0001,sigma2=vi,
            theta=yi,EhatQ=EhatQ)[1])}
        #confidence interval for between study variance based on chi−square
        #distribution with degrees of freedom equal to the estimate of the corrected
        #first moment to the approximate
        #the distribution of Q
        #function for calculation of upper bound
        f_upper_KDB <− function(g,sigma2,theta,EhatQ){
```

```
sum((theta-sum(theta/(sigma2+g))/sum(1/(sigma2+g)))^2/(sigma2+g))-qchisq
    (0.025,EhatQ)}
#calculation of upper bound
if(f_upper_KDB(ll,sigma2=vi,theta=yi,EhatQ=EhatQ)*f_upper_KDB(uu,sigma2=vi,
    theta=yi,EhatQ=EhatQ)<0){
tau2.ub_KDB <- as.numeric(uniroot(f_upper_KDB, c(ll, uu), tol = 0.0001,
    sigma2=vi,theta=yi,EhatQ=EhatQ)[1])}
#function for calculation of lower bound
f_lower_KDB <- function(g,sigma2,theta,EhatQ){
sum((theta-sum(theta/(sigma2+g))/sum(1/(sigma2+g)))^2/(sigma2+g))-qchisq
    (0.975,EhatQ)}
#calculation of  lower bound
if (f_lower_KDB(ll,sigma2=vi,theta=yi,EhatQ=EhatQ)*f_lower_KDB(uu,sigma2=vi,
    theta=yi,EhatQ=EhatQ)<0){
tau2.lb_KDB <- as.numeric(uniroot(f_lower_KDB, c(ll, uu), tol = 0.0001,
    sigma2=vi,theta=yi,EhatQ=EhatQ)[1])}
tau2 <- data.frame(tau2_KDB,tau2.lb_KDB,tau2.ub_KDB)
colnames(tau2) <- c("estimate", "ci.lb", "ci.ub")
rownames(tau2) <- c("tau2 (KDB)")
return(tau2)
}
```

G1.3 Function - metaSSWwithKDB

```
#Function for calculation of overall effect measure based on SSW
#User need to input values for effects yi,
#within-study variances vi,
#sample sizes nC and nT
#in control and treatment group
#and any estimator of between study variance - tau2
#required functions:metaSMDimprovedQ, ImprovedQmomentsSMD,
#parm, Qdelta, EQ, mom, cmom, EQ2
metaSSWwithKDB <- function(yi, vi, nC, nT){
        #estimation of between study variance
        tau2_estimates <- metaSMDimprovedQ(yi, vi, nC, nT)
        tau2 <- tau2_estimates[[1]]
        K <- length(yi)
        n=nC+nT
        nbar <- (nC*nT)/n
        thetabar_SSW <- sum(yi*nbar)/sum(nbar)
        varThetaBar_SSW <- sum((vi+tau2)*(nbar^2))/((sum(nbar))^2)
        Ltheta_SSW <- thetabar_SSW-qt(.975, df=K-1)*sqrt(varThetaBar_SSW)
        Utheta_SSW <- thetabar_SSW+qt(.975, df=K-1)*sqrt(varThetaBar_SSW)
        theta_estimates <- data.frame(thetabar_SSW, Ltheta_SSW, Utheta_SSW)
        names(theta_estimates) <- names(tau2_estimates)
        output <- rbind(tau2_estimates, theta_estimates)
        colnames(output) <- c("estimate","ci.lb","ci.ub")
        rownames(output) <- c("tau2_(KDB)","theta_(SSW_KDB)")
return(output)
}
```

G1.4 Function - metaHKSJwithKDB

```
#Function for calculation of overall effect measure
#based on HKSJ KDB
#User need to input values for effects yi,
#within-study variances vi,
#sample sizes nC and nT
#in control and treatment group
#and any estimator of between study variance - tau2
#required functions:metaSMDimprovedQ,ImprovedQmomentsSMD,
#parm,Qdelta,EQ,mom,cmom,EQ2
metaHKSJwithKDB <- function(yi,vi,nC,nT){
        #estimation of between study variance
        tau2_estimates <- metaSMDimprovedQ(yi,vi,nC,nT)
        tau2 <- tau2_estimates[[1]]
        K <- length(yi)
        thetabar_HKSJ <- sum(yi/(vi+tau2))/sum(1/(vi+tau2))
        w_HKSJ <- 1/(vi+tau2)
        var_HKSJ <- sum(w_HKSJ*(yi-thetabar_HKSJ)^2)/((K-1)*sum(w_HKSJ))
        Ltheta_HKSJ <- thetabar_HKSJ-qt(.975, df=K-1)*sqrt(var_HKSJ)
        Utheta_HKSJ <- thetabar_HKSJ+qt(.975, df=K-1)*sqrt(var_HKSJ)
        theta_estimates=data.frame(thetabar_HKSJ,Ltheta_HKSJ,Utheta_HKSJ)
        names(theta_estimates) <- names(tau2_estimates)
        output <- rbind(tau2_estimates,theta_estimates)
        colnames(output) <- c("estimate","ci.lb","ci.ub")
        rownames(output) <- c("tau2 (KDB)","theta (HKSJ KDB)")
return(output)
}
```

G1.5 Function - metaHKSJwithKDB

```
#required functions:Qdelta ,EQ,mom,cmom,EQ2,parm
ImprovedQmomentsSMD <- function(yi,vi,nC,nT){
        q<-(1-nT/(nT+nC))
        #inverse-variance weights
        w <- (vi)^(-1)
        ##total sample size
        N=nC+nT
        ###Calculation of corrected moments of Q distribution based on chi-square
            distribution proposed in a paper
        ###by Kulinskaya E, Dollinger M, Bjorkestol K Biometrics 2011
        nbar <- (nC*nT)/N
        Q <- numeric(0)
        EhatQ <- numeric(0)
        part <- numeric(0)
        par <- numeric(0)
        Qsum <- 0
        Q2sum <- 0
        EhatQsum <- 0
        E2hatQsum <- 0
        K <- length(N)
        par <- numeric(0)
        J <- exp(lgamma((N-2)/2)-lgamma((N-3)/2))/sqrt((N-2)/2)
        A <- 1/((nC*nT/N))          #### changed
        a <- (N-2)*J^2/(N-4)        #### changed
        B <- 1- 1/a
        g <- yi
        ########################################################################
        for (k in 1:K){
        par <- c(par,g[k],w[k],N[k],A[k], B[k])
        }
        pare <- numeric(0)
        Q <- Qdelta(par)[1]
        Qsum <- Qsum+Q
```

```
        Q2sum <- Q2sum+Q^2
        levelQ <- pchisq(Q,K-1,ncp=0,lower.tail=FALSE)
        gbar <- Qdelta(par)[2]
        wtm <- ((A+B*gbar^2))^(-1)
        for (k in 1:K){
        pare <- c(pare,gbar,wtm[k],N[k],A[k],B[k]) #estimated null parameters
        }
        mo <- mom(N,gbar*sqrt((nT*nC/N)),6)
        cmo <- cmom(N,q,mo,6)
        EhatQ <- EQ(pare,cmo)
        E2hatQ <- EQ2(pare,cmo)
        VARhatQ <- E2hatQ-EhatQ^2
        beta.est <- VARhatQ/EhatQ
        alfa.est <- EhatQ/beta.est
        moments <- data.frame(EhatQ,E2hatQ,VARhatQ,alfa.est,beta.est)
        colnames(moments) <- c("EhatQ","E2hatQ","VARhatQ","alfa","beta")
return(moments)
}
```

G1.6 Functions - parm,Qdelta,EQ,mom,cmom,EQ2 for KDB estimator

```
parm <- function(nC,nT,xC,xT,s){
        parm <- numeric(0)
        N <- nT+nC
        K <- length(N)
        J <- exp(lgamma((N-2)/2)-lgamma((N-3)/2))/sqrt((N-2)/2)
        A <- 1/((nC*nT/N))          #### changed
        a <- (N-2)*J^2/(N-4)        #### changed
        B <- 1- 1/a
        g <- J*(xT-xC)/s
        w <- (A+B*g^2)^(-1)
        for (k in 1:K){
        parm <- c(parm,g[k],w[k],N[k],A[k], B[k])
        }
return(parm)
}

mom <- function(N,nc,r){
        #calculates moments (at zero) of non-central t
        #nc=non centrality
        #r= the maximum moment required
        mom <- numeric(0)
        K <- length(N)
        nu <- N-2
        for (k in 1:r)
        {
                r1 <- floor(k/2)
                m0 <- 0
                for (j in 0:r1){
                        m0 <- m0+(choose(k,2*j)*(factorial(2*j)/(2^j*factorial(j)))*
                            nc^(k-2*j))
                }
                mo <- (nu/2)^(k/2)*exp(lgamma((nu-k)/2)-lgamma(nu/2))*m0
                mom <- c(mom,mo)
```

```
        }
        mom <- matrix(mom, nrow=K)
        return(mom)
}

EQ <- function(parm, cmo){
        m <- matrix(parm, nrow=5)
        g <- m[1,]
        w <- m[2,]
        B <- m[5,]
        U <- 1-w/sum(w)
        W <- sum(w)
        E1 <- cmo[,1]
        E2 <- cmo[,2]
        E3 <- cmo[,3]
        E4 <- cmo[,4]
        d1f <- -2*B*g*w^2
        d2f <- -2*B*w^2+8*B^2*g^2*w^3
        EQ <- sum(w*U*E2)+sum(U^2*d1f*E3)+sum((-U^2*d1f^2/W+U^2*d2f/2)*E4)-
        W^(-1)*(sum(U*d1f*E2))^2-W^(-3)*(sum (w*d1f*E2))^2-
        W^(-3)*(sum (w^2*E2))*sum((d1f^2-W*d2f/(2))*E2)+
        W^(-1)*sum((1-2*w/W+3*w^2/W^2)*d1f^2*E2^2)-2^{-1}*W^(-2)*sum(w^2*d2f*E2^2)
return(EQ)
}

Qdelta <- function(parm){
        m <- matrix(parm, nrow=5)
        g <- m[1,]
        w <- m[2,]
        W <- sum(w)
        gbar <- sum(w*g)/W
        Q <- sum(w*(g-gbar)^2)
return(c(Q, gbar))
}
```

```
cmom <- function(N,q, mom,r){
    #calculates central moments of perfectly corrected Cohen's g
    #is based on moments mom of non-central t with
    #non-centrality nc=sqrt(Nq(1-q))*delta
    mu <- mom[,1]
    K <- length(N)
    nT  <-  ceiling((1-q)*N)
    nC  <-  N-nT
    cmom <- numeric(0)
    J <- exp(lgamma((N-2)/2)-lgamma((N-3)/2))/sqrt((N-2)/2)
    for (k in 1:r)
    {
        cm0 <- 0
        for (j in 0:(k-1))
        {
            cm0 <- cm0+((-1)^j)*choose(k,j)*mu^j*mom[,k-j]
        }
        cm0 <- cm0+(-mu)^k
        cm0 <- (J/sqrt(nT*nC/N))^k*cm0
        cmom <- c(cmom,cm0)
    }
    cmom <- matrix(cmom,nrow=K)
    return(cmom)
}
```

```
EQ2 <- function(parm,cmo){
      m <- matrix(parm,nrow=5)
      g <- m[1,]
      w <- m[2,]
      B <- m[5,]
      K <- length(g)
      U <- 1-w/sum(w)
      W <- sum(w)

      d1f <-  -2*B*g*w^2
      d2f <-  -2*B*w^2+8*B^2*g^2*w^3



      d4Q2 <- 24*w^2*U^2
      d5Q2 <- 240*w*U^3*d1f
      d6Q2 <- 720*U^3*((U-2*w/W)*d1f^2+w*d2f)
      d4Q2ij <- 8*crossprod(t(w*U))+16*crossprod(t(w^2))/W^2
      diag(d4Q2ij) <- 0

      d5Q2ij <- 24*crossprod(t(U^2*d1f),t(w*U))+120*crossprod(t(U*w*d1f),t(w^2))/W
         ^2-48*crossprod(t(w^2*U),t(U*d1f))/W-
      48*crossprod(t(w^3),t(w*d1f))/W^3
      diag(d5Q2ij) <- 0

      d6Q2ij=(48/W^4)*
      (-2*W^3*crossprod(t(U^2*d1f^2),t(w))+8*W^2*crossprod(t(U*d1f^2),t(w^2))-18*W
         *crossprod(t(U*w*d1f^2),t(w^2))
      +W^4*crossprod(t(U*d2f),t(w))-W^3*crossprod(t(U*w*d2f),t(w))-W^3*crossprod(t
         (U*d2f),t(w^2))+6*W^2*crossprod(t(U*w*d2f),t(w^2))
      -8*W^3*crossprod(t(U^2*w*d1f),t(d1f))+8*W^2*crossprod(t(U*w*d1f),t(w*d1f))
         -24*W*crossprod(t(U*w^2*d1f),t(w*d1f))
      -2*W*crossprod(t(w^3),t(d1f^2))+3*crossprod(t(w^4),t(d1f^2))+W^2*crossprod(t
         (U*w^3),t(d2f)))
      diag(d6Q2ij) <- 0
```

```
d6Q2ijk <- (-16/W^4)*(W*crossprod(t(d1f^2),t(w^2))%o%w
+ W*crossprod(t(w^2),t(d1f^2))%o%w
+W*crossprod(t(w^2),t(w))%o%(d1f^2)
+  W*crossprod(t(d1f^2),t(w))%o%(w^2)
+ W*crossprod(t(w),t(d1f^2))%o%(w^2)
+W*crossprod(t(w),t(w^2))%o%(d1f^2)
-9*crossprod(t(d1f^2),t(w^2))%o%w^2
-9*crossprod(t(w^2),t(d1f^2))%o%w^2
-9*crossprod(t(w^2),t(w^2))%o%d1f^2
)+
(8/W^3)*(W*crossprod(t(d2f),t(w^2))%o%w
+W*crossprod(t(w^2),t(d2f))%o%w
+W*crossprod(t(w^2),t(w))%o%d2f
+W*crossprod(t(d2f),t(w))%o%w^2
+W*crossprod(t(w),t(d2f))%o%w^2
+W*crossprod(t(w),t(w^2))%o%d2f
-6*crossprod(t(d2f),t(w^2))%o%w^2
-6*crossprod(t(w^2),t(d2f))%o%w^2
-6*crossprod(t(w^2),t(w^2))%o%d2f
)-
(32/W^2)*(crossprod(t(U*d1f),t(U*d1f))%o%w*W
+crossprod(t(U*d1f),t(w*W))%o%U*d1f
+crossprod(t(w*W),t(U*d1f))%o%U*d1f
-6*crossprod(t(U*d1f),t(U*d1f))%o%w^2
-6*crossprod(t(U*d1f),t(w^2))%o%U*d1f
-6*crossprod(t(w^2),t(U*d1f))%o%U*d1f
+crossprod(t(w*d1f),t(w*d1f))%o%(w/W)
+crossprod(t(w*d1f),t(w/W))%o%(w*d1f)
+crossprod(t(w/W),t(w*d1f))%o%(w*d1f)
-12*crossprod(t(w*d1f),t(w*d1f))%o%(w^2/W^2)
-12*crossprod(t(w*d1f),t(w^2/W^2))%o%(w*d1f)
-12*crossprod(t(w^2/W^2),t(w*d1f))%o%(w*d1f)
+3*crossprod(t(d1f),t(d1f))%o%w^2
```

```
    +3*crossprod(t(d1f),t(w^2))%o%d1f
    +3*crossprod(t(w^2),t(d1f))%o%d1f
    )
    for (i in 1:K)
    {
        diag(d6Q2ijk[i,,]) <- 0
        diag(d6Q2ijk[,i,]) <- 0
        diag(d6Q2ijk[,,i]) <- 0
    }



    E2 <- cmo[,2]
    E3 <- cmo[,3]
    E4 <- cmo[,4]
    E5 <- cmo[,5]
    E6 <- cmo[,6]

    EQ2 <- sum(d4Q2*E4)/24+sum(d5Q2*E5)/120+sum(d6Q2*E6)/720+t(E2)%*%d4Q2ij%*%E2
      /8+t(E3)%*%d5Q2ij%*%E2/12+
    t(E4)%*%d6Q2ij%*%E2/48

    R <- 0
    for (i in 1:K){
        for (j in 1:K){
            for (k in 1:K){
                R <- R+d6Q2ijk[i,j,k]*E2[i]*E2[j]*E2[k]}}}

    EQ2 <- EQ2+R/48
    return(EQ2)
}
```
