# Supplementary material for: Estimation in meta‐analyses of mean difference and standardized mean difference
Source: Stat Med. 2019 Nov 11;39(2):171–91. doi: 10.1002/sim.8422 (PMC6916299; doi:10.1002/sim.8422)
Supplement: Supplementary file 1 — SIM_8422‐Supp‐0001.zip [file SIM-39-171-s001.zip › MD_SMD_WebAppendix_H0.pdf]

☐

APPENDIX

Web Appendix H

for

Ilyas Bakbergenuly, David C. Hoaglin, and Elena Kulinskaya

Estimation in meta-analyses of mean difference and standardized mean difference

SMD: Plots of bias and coverage for K=20

The point estimators of τ^2 are

- DL (DerSimonian-Laird)
- REML (restricted maximum likelihood)
- MP (Mandel-Paule)
- KDB (Kulinskaya-Dollinger-Bjørkestøl method)
- J (Jackson)

The interval estimators of τ^2 are

- QP (Q-profile interval)
- BJ (Biggerstaff and Jackson interval)
- PL (Profile likelihood interval)
- KDB (Kulinskaya-Dollinger-Bjørkestøl interval)
- J (Jackson interval)

The point estimators of δ are

- DL
- REML
- MP
- KDB
- J
- SSW (sample-size-weighted)

The interval estimators of δ are the companions to the inverse-variance-weighted point estimators

- DL
- REML
- MP
- KDB
- J

and

- HKSJ (Hartung-Knapp-Sidik-Jonkman interval)
- HKSJ KDB (HKSJ with KDB estimator of τ^2)
- SSW (SSW as center and half-width equal to critical value from t_{K-1} times estimated standard deviation of SSW with $\hat{\tau}^2 = \hat{\tau}^2_{KDB}$.

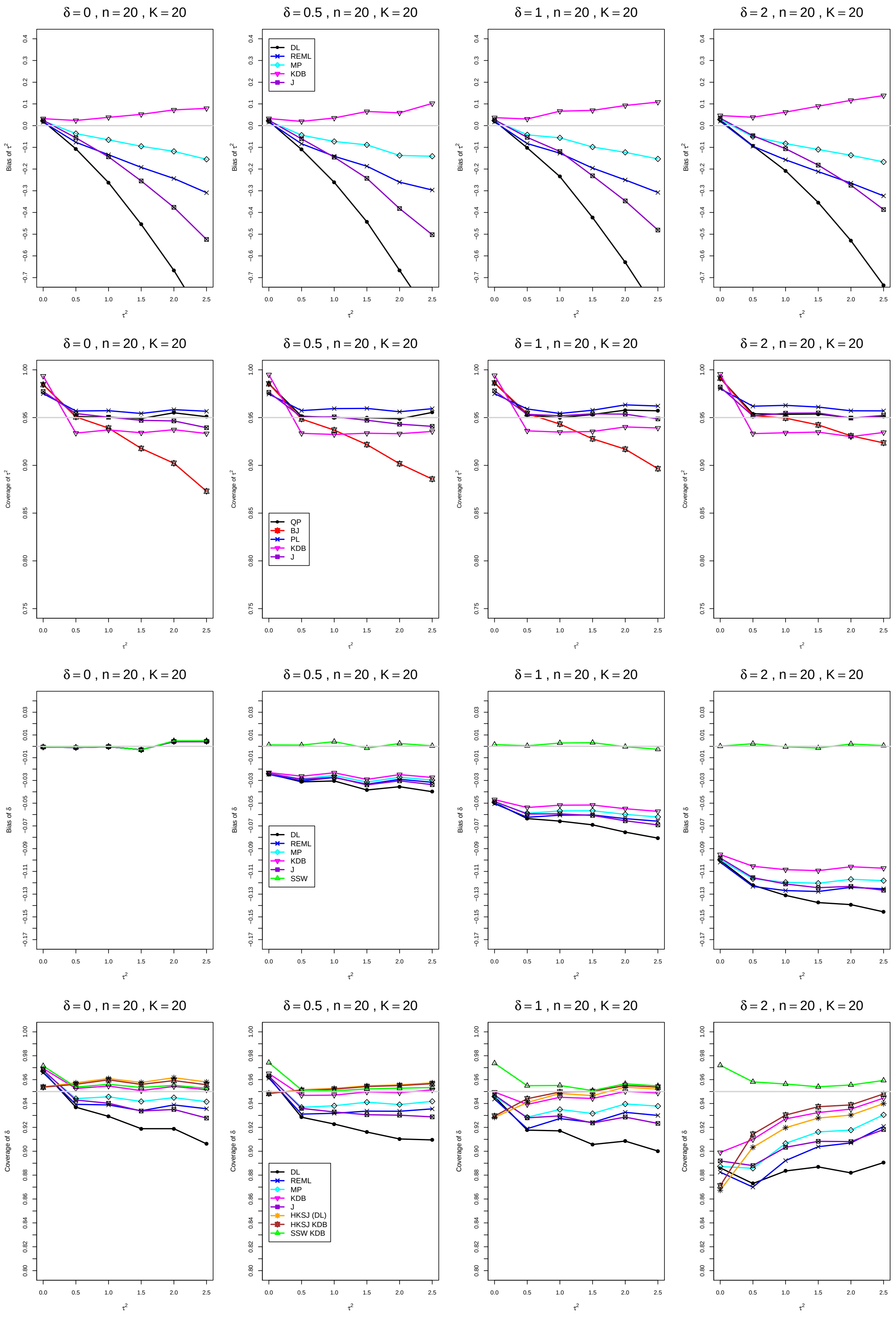

FIGURE H1 SMD: Performance of estimators of τ^2 and δ. $q = .5$, $n = 20$, and $\tau^2 = 0(0.5)2.5$.

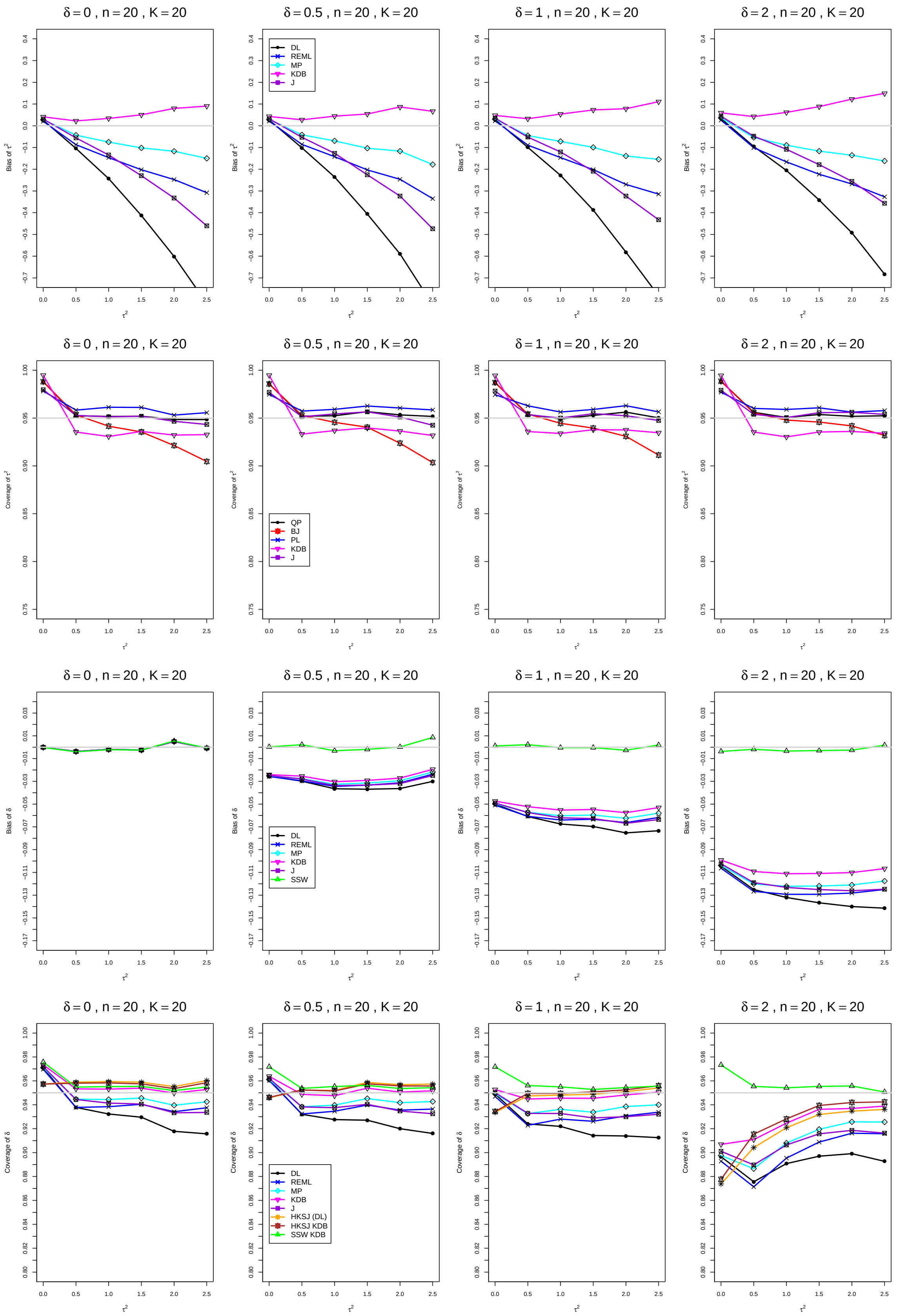

FIGURE H2 SMD: Performance of estimators of τ^2 and δ. $q = .75$, $n = 20$, and $\tau^2 = 0(0.5)2.5$.

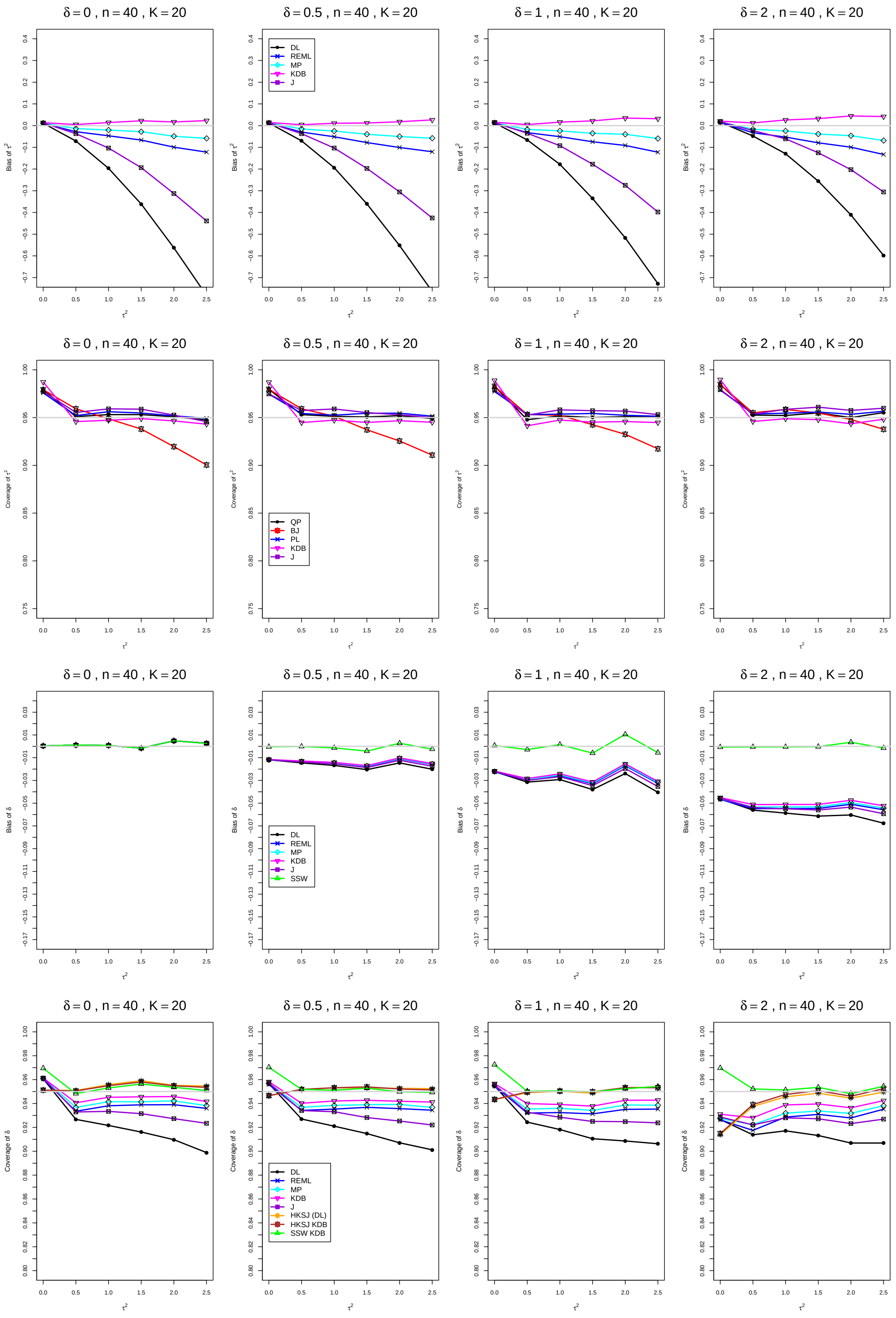

FIGURE H3 SMD: Performance of estimators of τ^2 and δ. $q = .5$, $n = 40$, and $\tau^2 = 0(0.5)2.5$.

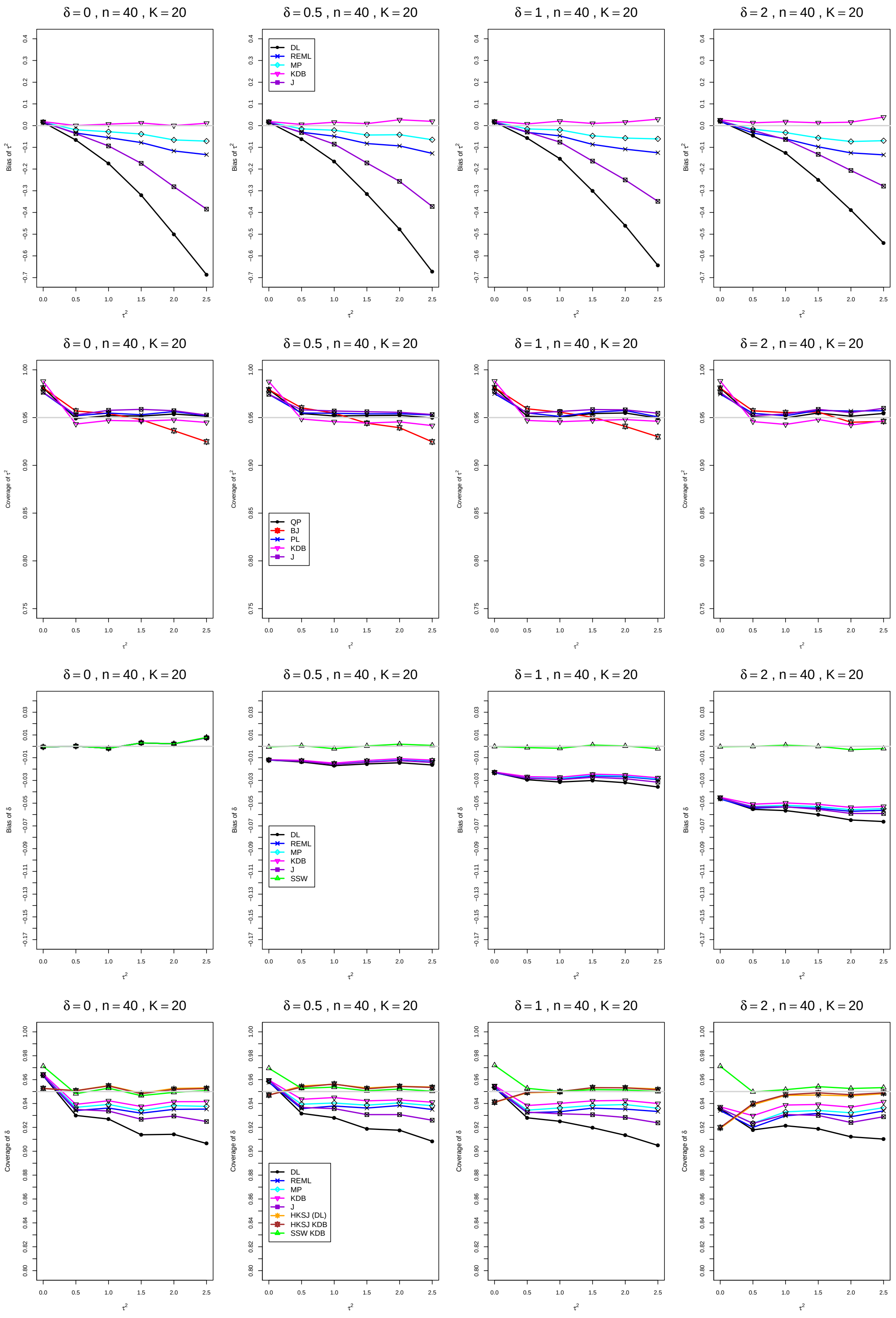

FIGURE H4 SMD: Performance of estimators of τ^2 and δ. $q = .75$, $n = 40$, and $\tau^2 = 0(0.5)2.5$.
